# Spatiotemporal variation in urban overheating magnitude and its association with synoptic air-masses in a coastal city

**DOI:** 10.1038/s41598-021-86089-2

**Published:** 2021-03-24

**Authors:** Hassan Saeed Khan, Mat Santamouris, Pavlos Kassomenos, Riccardo Paolini, Peter Caccetta, Ilias Petrou

**Affiliations:** 1 School of Built Environment, University of New South Wales (UNSW), Sydney, NSW 2052 Australia; 2 Department of Physics, University of Ioannina, 45110 Ioannina, Greece; 3 Data-61, The Commonwealth Scientific and Industrial Research Organization (CSIRO), Dick Perry Ave, Kensington, Perth, WA 6151 Australia

**Keywords:** Climate sciences, Environmental sciences, Energy science and technology, Engineering, Physics

## Abstract

Urban overheating (UO) may interact with synoptic-scale weather conditions. The association between meteorological parameters and UO has already been a subject of considerable research, however, the impact of synoptic-scale weather conditions on UO magnitude, particularly in a coastal city that is also near the desert landmass (Sydney) has never been investigated before. The present research examines the influence of synoptic-scale weather conditions on UO magnitude in Sydney by utilizing the newly developed gridded weather typing classification (GWTC). The diurnal, and seasonal variations in suburban-urban temperature contrast (ΔT) in association with synoptic-scale weather conditions, and ΔT response to synoptic air-masses during extreme heat events are investigated in three zones of Sydney. Generally, an exacerbation in UO magnitude was reported at daytime over the years, whereas the nocturnal UO magnitude was alleviated over time. The humid warm (HW), and warm (W) air-masses were found primarily responsible for exacerbated daytime UO during extreme heat events and in all other seasons, raising the mean daily maximum ΔT to 8–10.5 °C in Western Sydney, and 5–6.5 °C in inner Sydney. The dry warm (DW), and W conditions were mainly responsible for urban cooling (UC) at nighttime, bringing down the mean daily minimum ΔT to − 7.5 to − 10 °C in Western Sydney, and − 6 to − 7.5 °C in inner Sydney. The appropriate mitigation technologies can be planned based on this study to alleviate the higher daytime temperatures in the Sydney suburbs.

## Introduction

The global average temperatures have risen by 0.85 °C over the past century^1^ and adversely affecting outdoor thermal comfort. In addition to the temperature, other atmospheric variables including humidity, wind speed, atmospheric pressure, and cloud cover may synergistically interact to alter the thermal comfort. A significant variation in the frequency and magnitude of other atmospheric variables was also reported in recent decades. For instance, generally, a decline in wind speed was oberved^2^, whereas land-based precipitation was reported to have increased for the mid-latitudes due to higher evaporation rates (in connection to the higher temperatures), which has correspondingly increased the absolute moisture content^3^.

Globally, 55% of the world’s population is urbanite (2018 statistics), and the proportion is projected to increase to 68% by 2050^4^. Urban overheating (UO) is a local-scale phenomenon, where urban temperatures are comparatively higher than the surroundings due to the change in the urban surface characteristics (materials with higher heat storage capacity, and lower albedo), urban geometry, air pollution levels, anthropogenic heat fluxes, and climatic and the meteorological conditions^5,6^. The size of the city, population density, topography, and industrial development in the city also influence the UO magnitude^7^. Mostly, the strongest UO is reported at nighttime under calm (low wind speed), and clear sky conditions (cloudless sky)^8^. The daytime ΔT predominantly depends upon the urban–rural moisture contrast^9^, which is not only controlled by the land-use^10^, but also by synoptic-scale climatology^11^. Urbanization alters the radiative and aerodynamic characteristics of the surfaces, it changes the land–atmosphere heat and moisture exchange and modifies the atmospheric characteristics over the cities.

The large-scale weather conditions also influence the local-scale conditions and the interactions between both phenomena are interchangeable, where any of the conditions may prevail at different times or locations^12^. For instance, high-speed desert winds exacerbated the UO magnitude in Sydney^13,14^, and a decline in wind speed was also reported in China due to rapid urban growth^15,16^. The association between meteorological parameters and UO has already been studied extensively. For instance, regional wind speed and cloud cover have been concluded the most prominent meteorological parameters affecting ΔT by altering the ventilation and insolation conditions in the region^17^. As a rule of thumb, the regional wind speed and cloud cover are considered to be in an inverse relationship with UO, and the relational magnitude between UO and the meteorological parameters varies from place to place^18^. The enhanced regional wind speed due to secondary-air-circulation (wind flow from high-pressure rural zones to low-pressure urban zones) reduces the UO magnitude, depending upon the urban geometry^18–20^. Similarly, under cloudy conditions at nighttime, the longwave radiative losses at both urban and rural surfaces are reduced, which reduces the urban–rural thermal gradient. Whereas, the quick radiative cooling in rural areas and longwave emission in urban fabric from daytime heat storages exacerbate the urban–rural thermal contrast at nighttime under cloudless conditions^21–23^. The UO combined with large-scale synoptic/ meteorological conditions may also have a significant impact on human health, energy, the economy, and environmental quality^24^.

The trends in large-scale weather conditions and their connection with UO remained largely unexplored, particularly in a coastal city, which is also in the proximity of desert landforms (Sydney). The association between synoptic-scale conditions and UO had been investigated using various classifications including circulation-pattern-based classification (CPC)^18,25,26^, and multivariate weather-typing classification (WTC)^11,27,28^. Generally, in CPC, anticyclonic conditions are associated with the clear sky which brings undisturbed radiations^29^, whereas the cyclonic conditions are mostly connected with cloudy conditions^30^. While utilizing the CPC in Melbourne^18^, Buenos Aires^25^, Birmingham^26^, Poznan^17^, Debrecen^29^, Szeged^30^, and Athens^31^, anticyclonic conditions, low wind speed, and a lesser amount of cloud cover were linked with exacerbated UO. Contrarily, cyclonic conditions were largely connected to lower UO magnitude or with urban cooling (UC) in Poznan^17^, and Szeged^30^. While applying the multivariate WTC, generally dry weather types (WTs) in eastern USA^27^, and particularly dry tropical WT in Atlanta^11^, northeastern USA (Baltimore, Philadelphia, New York)^32^, and Phoenix^28^ were responsible for exacerbated nighttime UO. However, in northeast USA^32^, higher ambient temperatures at both urban and rural sites were also reported under moist WTs. In Atlanta^11^, lower mean ΔT was attributed to moist-polar WT. Further, the association between dry WTs and clear and calm meteorological conditions^28,32^, and moist WT and cloudy meteorological condition was also concluded^30^.

Extreme heat events are occurring with higher frequency, while a downward trend in extreme cold events is also reported^33^. Heatwaves (regional-scale phenomenon) coincide with UO and exacerbate the urban–rural temperature contrast as reported in studies conducted in Sydney and several other cities^13,14,34^. However, how the large-scale weather conditions are going to influence the local-scale conditions in a dualistically-influenced coastal city (Sydney), remains an open question. This study identifies the impact of synoptic-scale weather conditions on the spatial and temporal patterns of UO in Sydney. In addition to diurnal and seasonal variations in UO magnitude, the associations between local and synoptic-scale weather conditions are also investigated during extreme heat events. The results will help to design the appropriate schemes to attenuate the UO impact in the Sydney suburbs.

## Results

### Synoptic air-masses frequency

**Figure 1:**
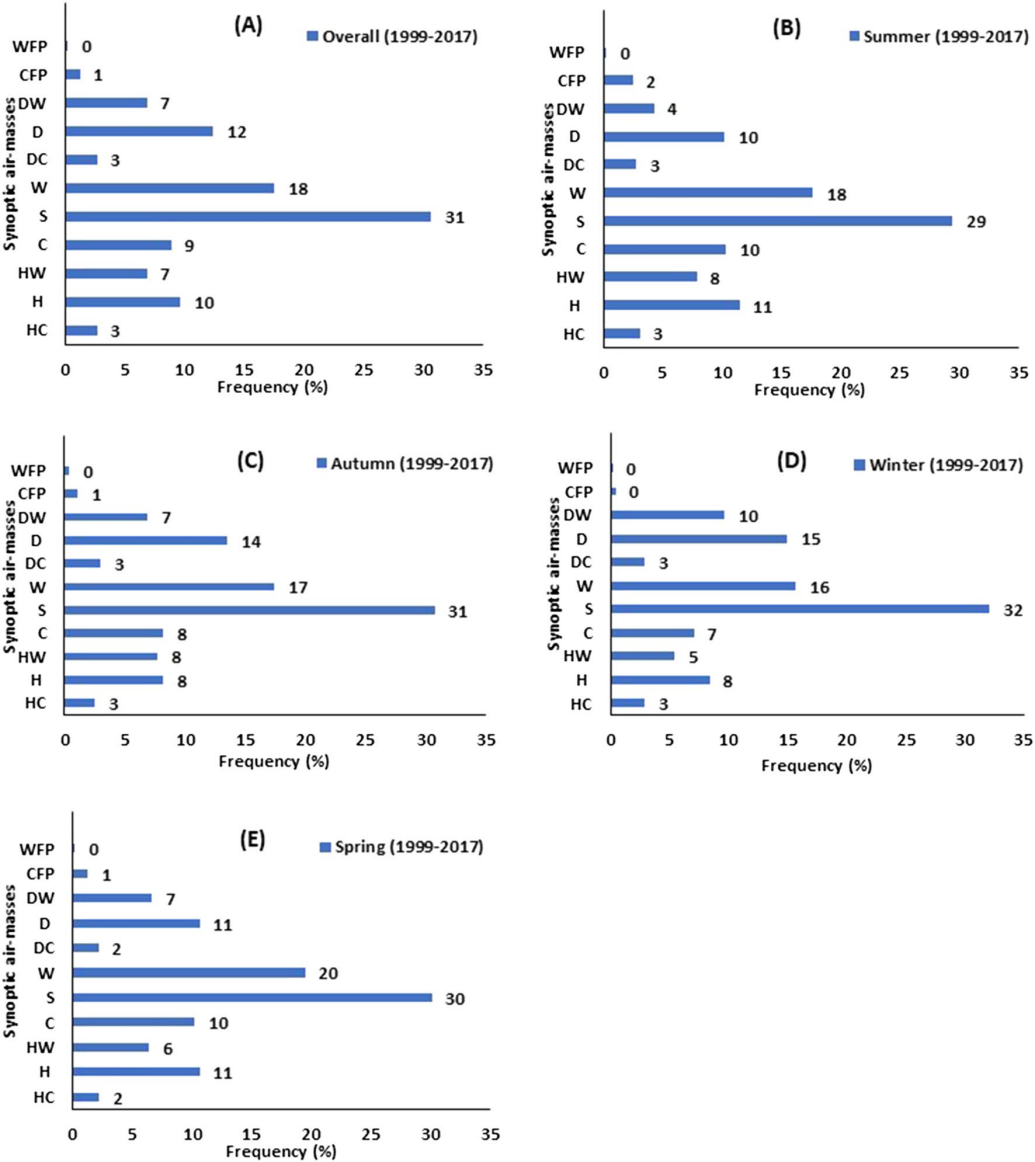
Synoptic air-masses Frequency from 1999 to 2017, (**A)** Overall, (**B)** Summer (DJF), (**C)** Autumn (MAM), (**D)** Winter (JJA), (**E)** Spring (SON).

The synoptic air-masses frequency was investigated in Sydney from 1999 to 2017. The seasonal (S) air-masses were the most frequent WT in all seasons (average around 31% of the time), followed by the warm air-masses (W: 18%), dry (D: 12%), humid (H: 10%), cold (C: 9%), dry warm (DW: 7%), humid warm (HW: 7%), humid cold (HC: 3%), dry cold (DC: 3%), and cold frontal passage (CFP: 1%) (Fig. 1).

The warm frontal passage (WFP) was almost ignorable in terms of frequency. The frequency of W, HW, H, and C WTs was comparatively higher during summer, and lower during winter. Contrarily, D and DW were occurring with lower frequency during summer, and with higher frequency during winter. The frequency of S WT was slightly reduced to 29% in summer, compared to all-seasons frequency (31%), whereas the W WT was comparatively higher in summer (18%), and in spring (20%). The HW WT frequency was surging during summer (8%), whereas dropping during winter (5%). The H WT was escalating to 11% during summer and spring from 8% during winter. The C WT frequency also increased to 10% during summer and spring, while during winter it was 7%. The DC and HC WTs were almost constant in all seasons (3%). The D WT were least frequent in summer (10%), and most frequent during winter (15%), compared to all-season (12%). Similarly, DW WT frequency reduced to 4% during summer, while increased to 10% during winter from 7% in all-seasons.

The variations in synoptic air-masses frequency were also examined over the years to understand which air-masses were occurring with higher frequency in recent years (Figure S1). The warm WTs were reported to occur with a higher frequency in recent years (2011–2017): DW (6–11%), W (14–19%), and HW (7–11%). Contrarily, some WTs were observed occurring least frequently in recent years: H (12- 8%), S (32–28%), DC (5–1%), and C (10–4%).

### Suburban-urban temperature difference (ΔT) frequency distribution

**Figure 2:**
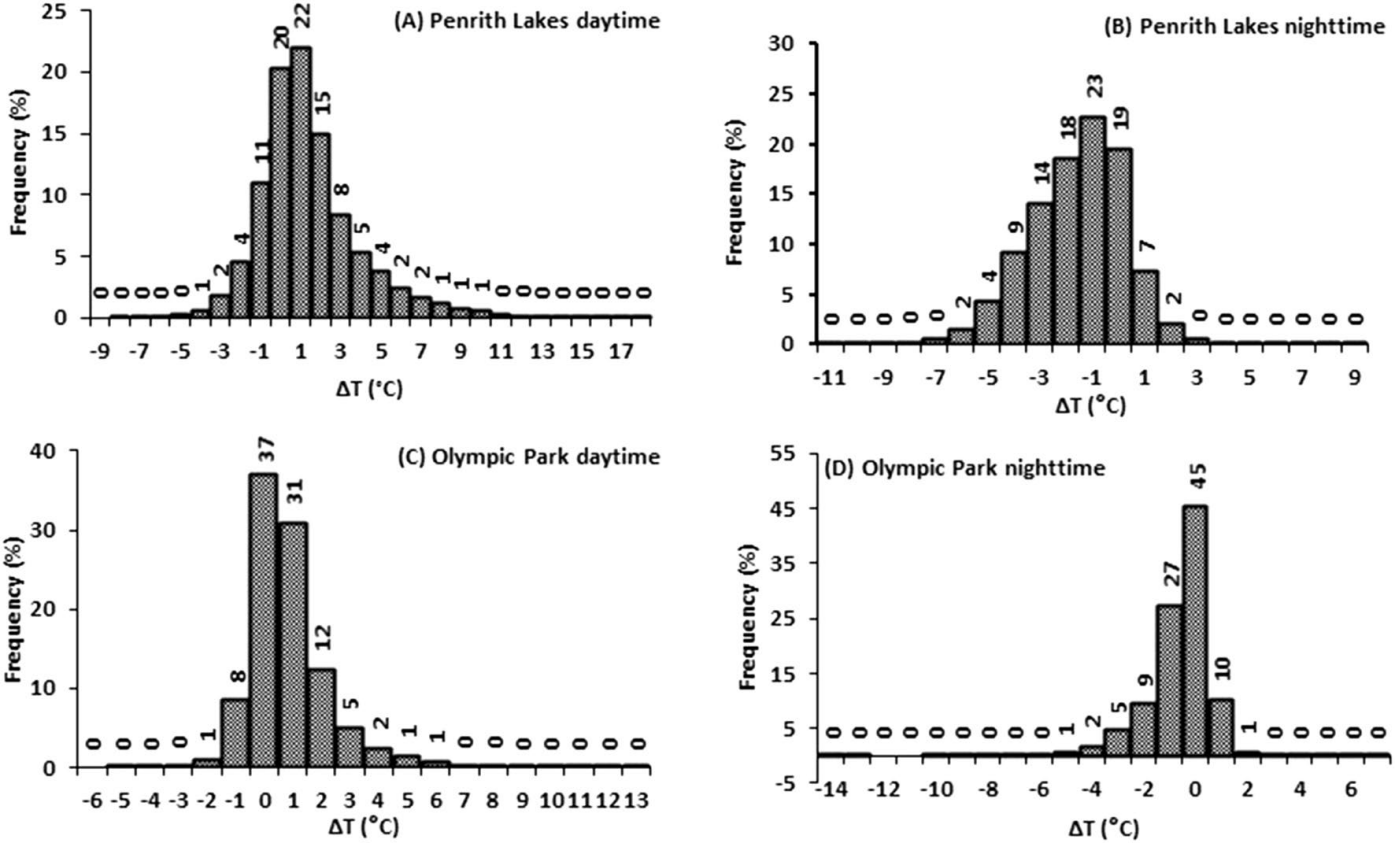
ΔT frequency distribution at different sites in Sydney at daytime and nighttime (**A)** Penrith Lakes at daytime, (**B)** Penrith Lakes at nighttime, (**C)** Olympic Park at daytime, (**D)** Olympic Park at nighttime.

The ΔT frequency distribution at daytime and nighttime was examined at all sites from 1999 to 2017 (2006-onward for Campbelltown, and 2013-onward for Liverpool) (Fig. 2 and S2).

In the daytime, ΔT (T_suburb_ – T _urb_) was reported positive for more than 50% (Penrith Lakes: 62%, Liverpool: 53%, Olympic Park: 53%) of the time in all suburbs except Campbelltown (46%), and Canterbury (43%). The daytime ΔT was exacerbating as the distance from the coast was increasing. The slightly lower frequency of positive ΔT during daytime at Canterbury is due to its least distance from the coast (7.5 m) compared to the other sites, whereas at Campbelltown, the highest tree canopy cover (38.5%), and more plantable surfaces are the major reasons (see “Data and methods”). Contrarily, at nighttime, the ΔT was reported negative for more than 90% of the time in all suburbs. The higher frequency of negative ΔT at nighttime can be attributed to a quick radiative cooling process due to the availability of higher nonurban surfaces in the suburbs compared to the Sydney CBD.

### Variations in ΔT magnitude over time

**Figure 3:**
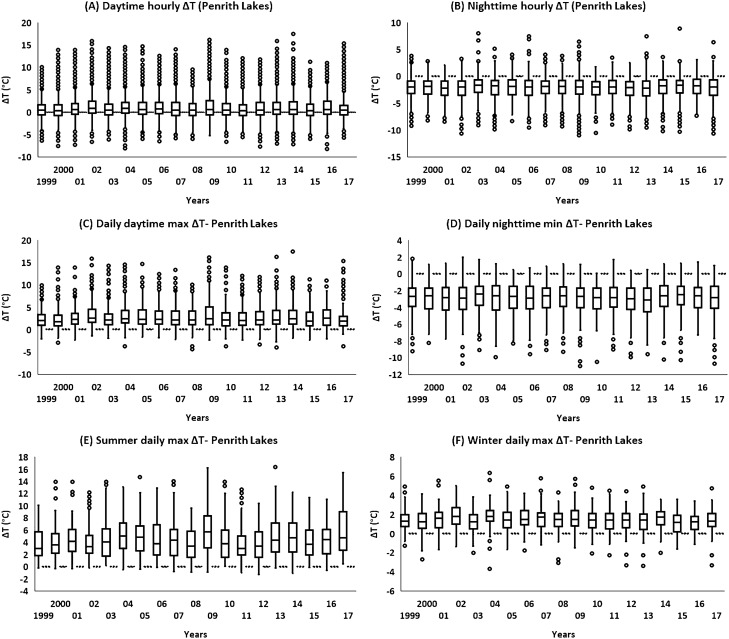
Variations in ΔT at Penrith Lakes over the time (1999–2017). (**A)** daytime hourly variations in ΔT, (**B)** nighttime hourly variations in ΔT, (**C)** Variations in daily max ΔT, (**D)** Variations in daily min ΔT, (**E)** Variations in daily max ΔT during summer, (**E)** Variations in daily max ΔT during winter. ΔT = 0 is illustrated by a dotted line. The boxplots are plotted according to the general convention (Q1-1.5*IQR and Q3 + 1.5*IQR), while the remaining dataset represents the outliers.

The variations in ΔT magnitude were also investigated over the years to comprehend the changes in ΔT magnitude with respect to time. The analyses were performed by utilizing the hourly ΔT (daytime, and nighttime), daily maximum and daily minimum ΔT, and daily max ΔT during various seasons. Generally, an increase in both hourly daytime ΔT (Fig. 3A, and S3), and daily maximum ΔT (Fig. 3C, and S5) was reported particularly after 2009.

For instance, the variations in mean daytime hourly ΔT before (1999–2008) and after 2008 (2009–2017) was from 0.97 to 0.99 °C at Western Sydney (Penrith Lakes), and from 0.31 to 0.51 °C at inner Sydney (Olympic Park). Similarly, the variations in mean daily max ΔT were from 2.83 to 2.89 °C at Western Sydney (Penrith Lakes), and from 1.45 to 1.72 °C at inner Sydney (Olympic Park) for the same duration.

Contrarily, a decrease in both hourly nighttime ΔT (Fig. 3B, and S4), and daily minimum ΔT (Fig. 3D, S6) was also noticed, and the drop in ΔT was even more noticeable after 2009. For instance, the decline in mean nighttime hourly ΔT before and after 2008 was from − 2.11 to − 2.13 °C at Western Sydney (Penrith Lakes), and from − 0.76 to − 1.41 °C at inner Sydney ( Olympic Park). Likewise, the drop in mean daily minimum ΔT before and after 2008 was from − 2.90 to − 2.93 °C in Western Sydney, and from − 1.30 to − 2.09 °C in inner Sydney. Seasonal ΔT (daily maximum) variations with respect to time were also examined, and in general, an increase in summer (Fig. 3E, and S7), and a decrease in winter (Fig. 3F, and S8) was reported particularly after 2009. For instance, the variations in mean daily maximum ΔT in summer before and after 2008 in Western Sydney were from 4.39 to 4.65 °C, whereas in inner Sydney, it was varying from 1.93 to 2.26 °C. The mean daily maximum ΔT in winter dropped from 1.47 °C (before 2008) to 1.3 °C (after 2008) in Western Sydney and 1.2 °C to 0.96 °C in inner Sydney for the same period.

### Synoptic air-masses and UO and UC magnitudes

To find an association between daily synoptic-scale WTs and UO, and UC, the upper 5% of daily maximum ΔT (UO) and lower 5% of daily minimum ΔT (UC) were computed at all sites. The threshold temperatures for UO and UC are shown in Table 1. The threshold temperatures for UO were higher in Western Sydney suburbs, and lower in inner Sydney suburbs. The threshold temperatures for UC were varying at various Sydney sites according to the site-characteristics.

**Table 1:** Threshold temperatures for the upper 5% ΔT (UO) and lowest 5% ΔT (UC) at all Sydney sites.

	ΔT (Penrith Lakes—OBS Hill) (°C)	ΔT(Campbelltown-OBS Hill) (°C)	ΔT(Liverpool—OBS Hill) (°C)	ΔT (Olympic—OBS Hill) (°C)	ΔT(Canterbury—OBS Hill) (°C)
95th percentile of daily maximum temperature	8.2	6.4	6.2	4.9	3.8
5th percentile of daily minimum temperature	− 6.5	− 8.45	− 6.1	− 4.7	− 6.6

**Figure 4:**
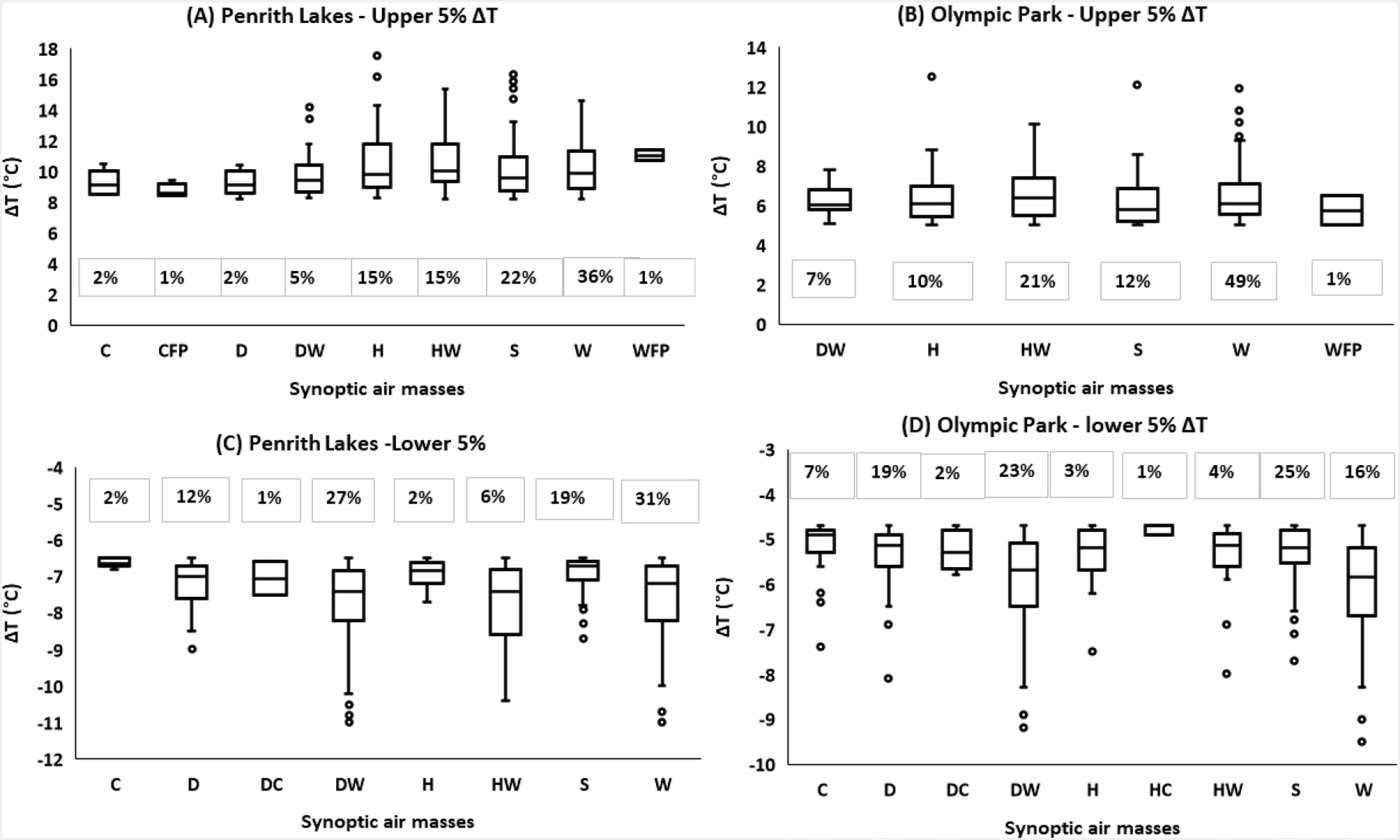
Higher 5% (UO) and lower 5% (UC) ΔT at different Sydney sites under different synoptic air-masses. **(A)** UO at Penrith Lakes, **(B)** UO at Olympic Park **(C)** UC at Penrith Lakes, **(D)** UC at Olympic Park. The boxplots are plotted according to the general convention (upper and lower extremes: Q1-1.5*IQR and Q3 + 1.5*IQR).

While investigating the impact of synoptic air-mass on UO magnitude, HW, W, and H WTs were reported as the most dominant weather conditions exacerbating the UO magnitude (Fig. 4A,B and S9A–S9C). Further, W WT was observed occurring with higher frequency, followed by HW, S, and H conditions (80–90% of the time). However, in inner Sydney W conditions, whereas in Western Sydney, HW conditions were more governing. At Western Sydney sites, W conditions occurred for approximately 38% of the time, while HW and H conditions occurred for 17%, and 15.5% of the time respectively. The mean value of the UO magnitude at various Western Sydney sites ranged between 8 and 10.5 °C (max: 13–17 °C), during these dominant WTs (H, HW, and W), slightly higher during HW and H conditions. At inner Sydney sites, W conditions occurred for 48.5% of the time, followed by HW (20%), and H (11%) conditions. The mean value of UO magnitude in inner Sydney during these dominant WTs was between 5–6.5 °C (max: 10–12 °C), comparatively higher during W conditions.

The slightly higher UO magnitude at Western Sydney sites during HW and H conditions is due to the reduced evaporation/evapotranspiration potential in the western suburbs due to higher ambient humidity, despite having a higher percentage of potentially plantable surfaces. HW conditions in the Sydney region could be attributed to tropical maritime Tasman air-masses (warm, moist, and unstable) coming from the north of the Tasman sea. The H condition could be attributed to the temperate maritime weather patterns, bringing very moist air from the sea, while due to the blue mountains in the west, westerly Fohn-like winds on the leeward side may increase the temperatures in western and inner Sydney through adiabatic warming. Conversely, UO magnitude at inner Sydney sites was comparatively more influenced by the W conditions. The tropical continental air-masses might be responsible for W, and DW conditions in the region, arise over central Australia, and are very hot, dry, and unstable. The higher the distance from the coast, the sites are more affected in terms of UO magnitude. The H conditions in inner Sydney sites are less influential due to the higher mixing of the coastal winds (due to the lower distance from the coast, compared to Western Sydney).

While examining the association between UC magnitude and synoptic-scale weather conditions (Figs. 4C, D, and S9D-S9F), it was observed that in terms of WT frequency, H, and HW WTs (during UO) were replaced by the D and DW WTs. H and HW WTs frequency were reduced (UO to UC) from 12 to 2%, and 20 to 5% respectively, while DW and D WTs frequency was increased from 6 to 31%, and from an insignificant value to 16% respectively. The D, DW, and W conditions occurred around 70–80% of the time, in association with UC. However, in terms of UC magnitude, W, DW, and HW were the most dominant synoptic conditions, reducing the temperatures in the suburbs. The mean UC magnitude during the dominant WTs (almost the same during W, DW, and HW) was between − 7.5 to − 10 °C (min: − 11 °C to − 14.5 °C) in Western Sydney sites, while in inner Sydney sites, it varied between − 6.0, and − 7.5 °C (min: − 9.5 °C to − 11.0 °C). HW and W conditions were slightly more dominant in terms of UC magnitude in Western Sydney, while W and DW were comparatively more noticeable in inner Sydney.

From the results (UO/UC association with synoptic-scale weather conditions, and ΔT frequency at daytime and nighttime), it can be presumed that these extreme UC cases occurred at nighttime, as almost the same warmer conditions (HW, W, and DW) were responsible for both UO and UC magnitudes, and 80–90% of the time UC phenomenon was taking place at nighttime. Thus, the association between diurnal ΔT variations (daily daytime max ΔT, and daily nighttime min ΔT), and daily synoptic-scale WTs were examined.

### Synoptic air-masses and diurnal variation in daily daytime maximum (max) ΔT and daily nighttime minimum (min) ΔT

**Figure 5:**
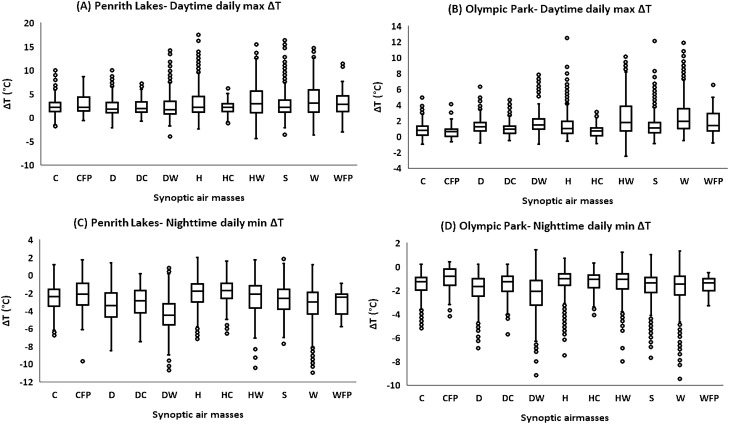
Daytime daily maximum ΔT and nighttime daily minimum ΔT at different Sydney sites under different synoptic air-masses. (**A)** Daytime daily max ΔT at Penrith Lakes, (**B)** Daytime daily max ΔT at Olympic Park, (**C)** Nighttime daily minimum ΔT at Penrith Lakes, (**D)** Nighttime daily minimum ΔT at Olympic Park. The boxplots are plotted according to the general convention (upper and lower extremes: Q1-1.5*IQR and Q3 + 1.5*IQR).

The associations between synoptic-scale WTs, and daily daytime max ΔT (Fig. 5A,B, and S10A-S10C), and daily nighttime min ΔT (Fig. 5C,D, and S10D-S10F) were examined. In Western Sydney, H, HW, and W WTs were mainly responsible for higher daytime ΔT, while in inner Sydney HW, and W WTs were intensifying the daytime ΔT. The H, HW, and W WTs accounted for 35% of the time: H(10%), HW (7%), and W(18%) (Fig. 1). The mean daytime max ΔT during HW and W conditions was between 2.8 and 3.8 °C at Western Sydney sites (almost the same under both conditions). During the H condition, the mean daytime max ΔT was comparatively lower. In inner Sydney, the mean daytime max ΔT was almost the same for both dominant WTs (HW, and W) and ranged between 2.0 and 2.4 °C at various sites. The lesser the distance from the coast, ΔT was accordingly lower.

At nighttime, instead of H, and HW conditions, the D and DW conditions along with W conditions were responsible for nighttime min ΔT. The W, DW, and D conditions occurred for 37% of the total time: W(18%), DW (7%), D (12%) (Fig. 1). Among these three WTs, DW conditions were mainly responsible for minimum nighttime ΔT. The mean nighttime min ΔT during DW condition was − 4 °C to − 6.5 °C in Western Sydney and − 2.5 to − 4.5 °C in inner Sydney sites. The mean nighttime min ΔT during other dominant WTs (D, and W) was comparatively low: − 3 °C to − 5 °C in Western Sydney and − 2.0 °C to − 3.3 °C in inner Sydney.

The HW and H conditions were responsible for higher daytime ΔT since the latent heat flux was less effective in the suburbs in the presence of higher ambient moisture, and a significant amount of available energy was partitioning into sensible heat flux, which was increasing the temperatures in the suburbs (particularly in Western Sydney). Contrarily, being under the influence of coastal winds, the Sydney CBD was exhibiting relatively lower daytime temperatures, compared to inner and Western Sydney. Further, urban shading might be another factor of lower daytime temperatures in the Sydney CBD. During the W conditions, the high-speed desert winds (tropical continental) might be responsible for higher daytime ΔT (advection from the hot source in the suburbs), which not only increases the ambient temperatures but also affect the latent heat flux potential in the region by sweeping the site ambient moisture. At nighttime, DW, W, and D conditions were more dominant, which again might originate from the continental side of the city. At nighttime, advection from the desert side has a lesser effect due to low-wind speed. The calm winds and cloudless sky conditions at nighttime during such dry WTs may enhance the radiative cooling process in the suburbs (lower ambient temperature), while more longwave radiations emission (attributed to higher daytime storages in the urban surface), and urban shading in Sydney CBD might be responsible for amplified nocturnal temperatures in Sydney CBD, which results in a daily minimum ΔT.

### Synoptic air-masses and Seasonal variations in daily max ΔT

**Figure 6:**
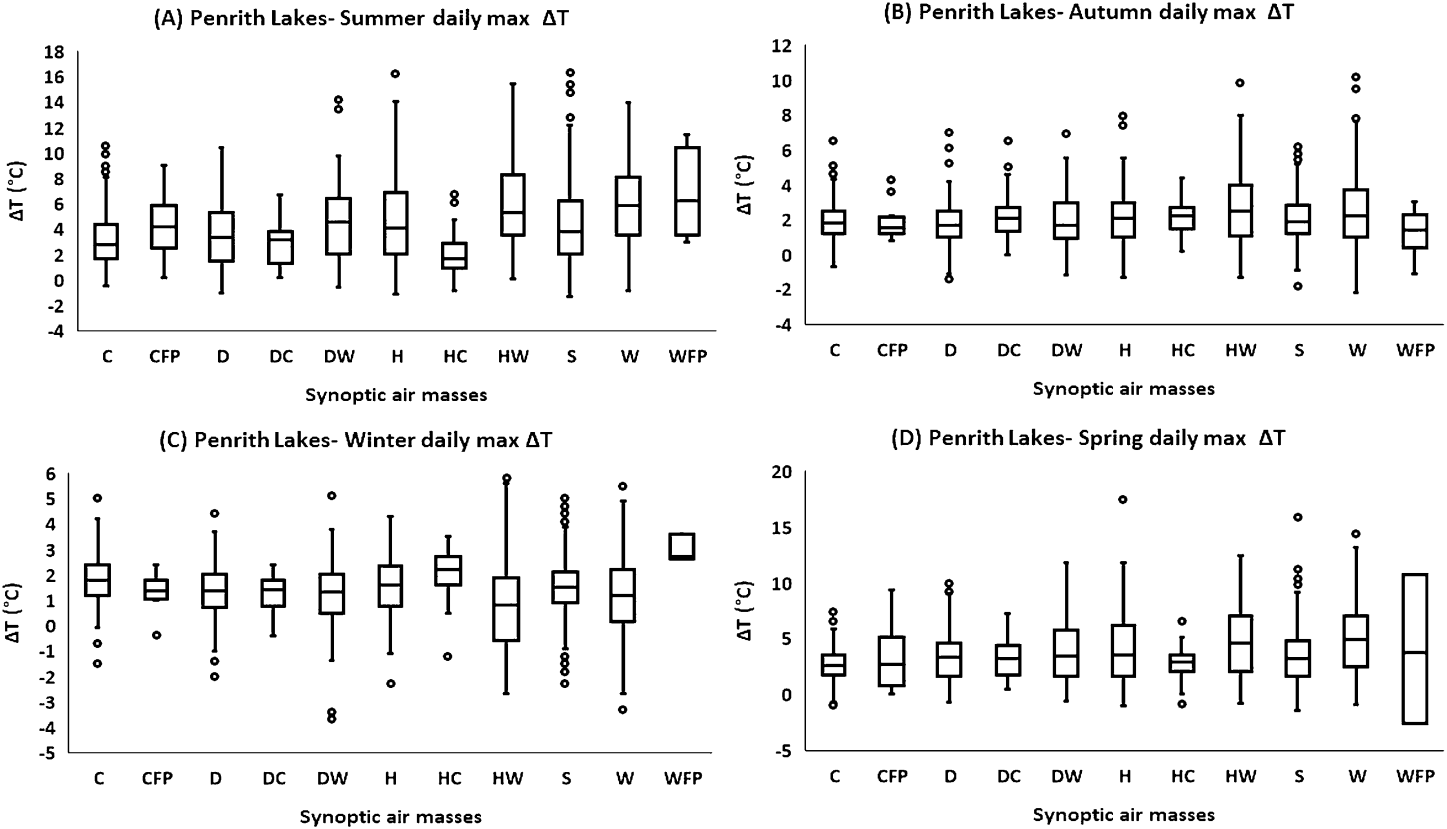
Synoptic air-masses and seasonal daily max ΔT comparison (1999–2017). (**A)** Penrith Lakes during summer, (**B)** Penrith Lakes during Autumn, (**C)** Penrith Lakes during winter, (**D)** Penrith Lakes during spring. The boxplots are plotted according to the general convention (upper and lower extremes: Q1-1.5*IQR and Q3 + 1.5*IQR).

The association between seasonal variations in daily max ΔT and synoptic air-masses was also investigated. In summer (Figs. 6A, S11), and autumn (Figs. 6B, S12), HW, and W conditions were the most dominant at all sites, magnifying the daily maximum ΔT. The HW and W conditions are referring to the dualistic synoptic systems, available on the opposite sides of the city; one originating from the coastal side of the city, while the other from the continental side. The HW and W conditions occurred for 25% of the total time (HW: 7.9%, W: 17.7%) during summer and autumn (Fig. 1B,C). During the summer, the mean daily max ΔT under HW and W conditions was between 4.5 and 6.0 °C in Western Sydney sites, and 2.8–3.5 °C in inner Sydney sites, slightly higher under HW condition. The mean daily max ΔT in autumn under dominant WTs (HW, W) was between 2.0 and 2.6 °C in Western Sydney sites, and 1.4–2.0 °C in inner Sydney sites, slightly higher under HW conditions.

In spring HW, W, and H conditions in Western Sydney, and HW, and W conditions in inner Sydney were predominant. The mean daily max ΔT at Western Sydney suburbs was slightly higher under HW, and W conditions, compared to H conditions. The HW and W conditions occurred for 27% (HW: 6.4%, W: 20%) of the total springtime (Fig. 1E). The mean daily max ΔT was varying between 3.6–4.8 °C in Western Sydney, and 2.4–3.2 °C in inner Sydney (almost the same during both HW and W conditions) (Fig. 6D, and S14).

In winter, although the highest daily maximum ΔT was also reported under HW, and W conditions at all sites, however, in Western Sydney, the mean ΔT (daily max) was also elevated under HC, C conditions. The HC and C conditions occurred for 10% (HC: 2.9%, C: 7.1%) of the total wintertime, while W and HW occurred for 21% of the wintertime (W: 15.7%, HW: 5.5%) (Fig. 1D). The mean daily max ΔT in Western Sydney suburbs ranged between 0.6 and 2.0 °C, relatively higher during HC conditions, followed by C, W, and HW conditions (Fig. 6C, and S13). The H and S conditions were also responsible for magnified ΔT in Western Sydney during winter. However, in inner Sydney W, DW, and HW conditions were mainly responsible for amplified daily max ΔT. Besides, D and S conditions were also influential in inner Sydney during winter. The mean daily max ΔT was fluctuating between 1.0 and 1.3 °C in inner Sydney sites under HW, W, and DW conditions (almost the same during all dominant WTs). The HC conditions might be attributed to southern maritime air-masses which produce cloudy weather accompanied by light precipitation in Sydney during winter.

### Synoptic air-masses and daily max ΔT during heatwaves and non-heatwaves

**Figure 7:**
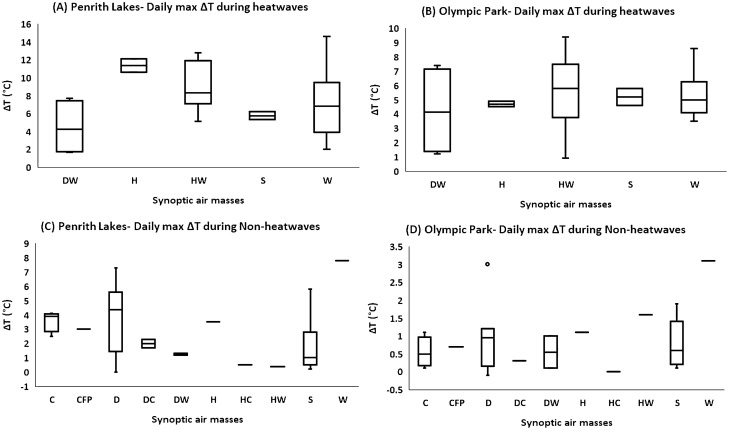
Synoptic air-masses and *daily max* ΔT during heatwaves and non-heatwaves). (**A)** Penrith Lakes during heatwaves, (**B)** Olympic Park during heatwaves, (**C)** Penrith Lakes during non-heatwaves, (**D)** Olympic Park during non-heatwaves. The boxplots are plotted according to the general convention (upper and lower extremes: Q1-1.5*IQR and Q3 + 1.5*IQR).

UO response to daily synoptic-scale WTs was also investigated during heatwaves and non-heatwaves. Firstly, the daily synoptic-scale WTs frequency during heatwaves and non-heatwaves was investigated. During heatwaves, HW, and W WTs occurred more frequently, approximately 76% of the total time (HW: 52%, W: 24%), followed by DW (12%), H( 6%), and S (6%) conditions (Figure S15A). During non-heatwaves, the most prominent conditions were S(34%), D(25%), and C(13%) (Figure S15B). The event-wise synoptic airmasses frequency was also investigated and it was observed that in recent years (2009–2017), HW WT frequency had increased during heatwaves (Figure S15C), while during non-heatwaves, D conditions were observed more frequently (Figure S15D). The HW and W conditions were also responsible for exacerbated UO magnitude during heatwaves at all Sydney sites. The HW conditions were comparatively more aggressive, and the mean UO magnitude ranged between 6 and 9 °C at Western Sydney sites, and 3.8–5.4 °C in inner Sydney sites under dominant WTs (HW, and W) (Figs. 7A,B, S16A-S16C). During non-heatwaves, D conditions in Western Sydney, and D and S conditions in inner Sydney were more presiding in terms of UO magnitude (Fig. 7C,D, S16D-S16F). The mean UO in Western Sydney during non-heatwaves under D conditions ranged between 2.4 and 4.0 °C, while the mean UO in inner Sydney during non-heatwaves under D and S conditions varied between 0.6 and 1.0 °C (almost the same during both WTs).

## Discussion

The land-atmospheric interaction (energy and moisture transfer) is significantly affected by urbanization. Sydney is growing towards the west/southwest, and the rapid urbanization is influencing the atmospheric circulations. The interactions between synoptic-scale weather conditions and UO magnitude were investigated in Sydney and warm WTs (DW, W, and HW) were reported occurring with higher frequency, particularly in recent years. Specifically, a significant increase was noted in HW and W conditions frequency, especially during summer months and during extreme heat events (heatwaves). These results are consistent with the study carried out in North America, where generally warm WTs were observed with higher frequency, while a drop in the cool WTs was also reported^35^. In North Carolina, summer months were also dominated by the moist tropical (HW) WTs^36^.

The UO magnitude in Sydney was exacerbating as the distance from the coast was increasing, despite having a higher proportion of tree canopy cover and non-urban surfaces in inner and Western Sydney. The same results were also reported in Los Angeles where higher thermal unbalance was observed for inland sites^37^. The amplified UO was noted at daytime under H, HW, and W conditions (almost in all seasons and during heatwaves). The enhanced moisture content in the air under moist WTs (HW conditions- attributed to tropical maritime Tasman air-mass/ H conditions-attributed to temperate maritime weather pattern) may keep the suburbs (inland sites) warmer at the daytime by reducing the latent heat flux potential in the region. Similarly, advection from the high-speed desert winds (warm air-attributed to tropical continental airmass) under W conditions also amplifies the daytime ambient temperatures at inland sites. On the other hand, the lower daytime temperatures in the Sydney CBD (located near the coast) can be attributed to the coastal-effect (maintained temperatures due to steady sea breeze), and urban shading. The lower urban temperatures during the early day were also linked to urban shading in Salamanca^38^. These results are consistent with the study carried out in New York, where higher daytime temperatures were noted at rural sites during moist WTs, compared to the urban areas (UC at daytime)^32^.

The higher UC magnitude was noted at nighttime under DW, W, and D conditions. Under dry conditions (cloudless sky and calm winds), suburbs (inland sites) cool faster due to radiative cooling, compared to the Sydney CBD where the longwave radiation emission from daytime heat storages amplifies the nocturnal temperatures. Further, the urban shading may also decelerate the cooling process in the Sydney CBD as reported in another study^39^. These results are consistent with the studies carried out in Atlanta^11^, and the northeast USA (New York, Philadelphia, and Baltimore )^27,32^, where higher urban–rural temperature contrast (UHI) was noted at nighttime under dry and hot conditions. The higher nighttime urban–rural thermal contrast (UHI = T_urb_- T_rural_) in Atlanta and other US cities and higher nighttime UC (T_suburbs_ – T _Urb_) in Sydney is due to different methods of ΔT calculation.

Previously, exacerbated UO magnitude was reported at nighttime under favorable synoptic-scale weather conditions in different cities around the globe^17,25,27,32^. In contrast to those studies, daytime ΔT was reported positive for 50–62% of the time (all seasons) in three zones of Sydney, while around 90% of the time, nighttime ΔT was recorded negative at all sites. The location of the city near the coast (reference station), urban shading, and the prevailing synoptic-scale weather conditions might be the primary reasons behind the different results. A positive daytime ΔT was also reported in some other coastal cities including Athens^34^, and Los Angeles^37^, except Melbourne and Adelaide^40^ where higher nighttime UO magnitude was reported due to inland site selection.

Under favorable conditions(HW, W, H), the mean daily max UO magnitude was reaching to 8–10.5 °C (max: 13–17 °C) in Western Sydney, whereas in inner Sydney 5–6.5 °C (max: 10–12 °C) was recorded. The magnitude of UO in Sydney is quite high, compared to the previous studies where under hot and dry weather conditions max daily mean ΔT was reported 3.84 °C in Atlanta^11^, and 3.5 °C in New York^32^, while under anticyclonic conditions 2.8 °C in Buenos Aires^25^, 3.6 °C in Melbourne^18^, and 1.2 °C in Poznan^17^ was noted. The mean daily max ΔT (2.5 °C) in Birmingham^26^ was also associated with the anticyclonic conditions. Mostly, these anticyclonic conditions were linked to calm winds and cloudless sky conditions (dry conditions), and were exacerbating the UO at nighttime as explained earlier. The urbanization impact in Western Sydney is more prominent due to the presence of the dualistic synoptic system, available on the opposite sides of the city as concluded in studies^13,14^, while investigating the interactions between local-scale (UO) and regional-scale (heatwaves) phenomena. The impact of the dualistic synoptic systems is also quite evident in the present study as during heatwaves, and in extreme UO cases, W, and HW conditions were not only occurring frequently but were also responsible for higher UO magnitude.

### Implication

The occurrence of warm air-masses (HW, and W) with higher frequencies (especially in summer, and during extreme heat events), and the rapid urbanization in Western Sydney is worrisome particularly in terms of human health. The low-income population, aged people, and people with pre-existing health conditions would be at higher thermal risk, which may increase the mortality and morbidity rates, and will be an added burden on the health sector. In New York^41^, and Rome^42^, DW and HW, while in Shanghai, HW conditions were associated with higher mortality and morbidity rates during summer. Further, it will also have adverse impacts on energy consumption and environmental quality as under HW/ W conditions in summer and during extreme heat events, the anthropogenic flux will also increase further due to the higher usage of airconditioning devices^43,44^, which will further deteriorate the environmental quality (due to the increased ozone formation^45^), and exacerbate the UO magnitude. A projected increase in extreme heatwave events^46^, and a projected increase in the proportion of urbanites living in Australian capital cities^47^ will make the Sydneysiders further vulnerable. The higher daytime temperature in Western Sydney can be alleviated by installing material/ systems retaining higher moisture content, including green roofs^48^, more vegetated surfaces, and by using the cool materials in the urban fabric^49^.

## Conclusion

An association between ΔT and daily synoptic-scale weather conditions was investigated in the greater Sydney region. The warmer air-masses (DW, HW, W) were reported to occur with a higher frequency, particularly in recent years. The W and HW WTs were noticed occurring with higher frequency in summer and during extreme heat events. UO was primarily reported in the daytime (more than 50% of the time), while UC was largely observed at nighttime (more than 90% of the time). Further, UO magnitude was found exacerbating at daytime as the distance from the coast was increasing, despite having higher tree canopy cover, and potentially plantable surfaces in the suburbs. The HW, W WTs were mainly responsible for exacerbated UO magnitude at daytime in all seasons and during extreme heat events, while UC at nighttime was mostly attributed to DW and W conditions. In Western Sydney, UO magnitude (mean daily max ΔT) was reported 8–10.5 °C (max: 13–17 °C) under HW, and W conditions, whereas in inner Sydney it was 5–6.5 °C (max: 10–12 °C). The higher UC magnitude (mean daily min ΔT) was reported − 7.5 to − 10 °C (min: − 11 to − 14.5 °C) in Western Sydney, and − 6 °C to − 7.5 °C (min: − 9.5 °C to − 11.0 °C) in inner Sydney primarily under DW and W conditions. The synoptic-scale weather conditions in the form of dualistic-circulation (HW, and W) are influencing the local-scale conditions in Sydney, and adversely affecting the daytime temperatures in the suburbs. Appropriate mitigation technologies should be engineered to enhance the coastal wind penetration in the suburbs and to reduce the circulation of the continental winds.

## Data and methods

### Climate and geographical location

The greater Sydney region is situated along the coastline of the South Pacific Ocean and has a humid subtropical climate (cfa classification under the Koppen-Geiger climate)^50^. Sydney is geographically the largest city in Australia, having 12,367.7 km^2^ land area and is extended by 70 km from the eastern coastline to the Blue mountain in the west^51^. Sydney's central business district (CBD) is located near the coastline and is mostly under the impression of coastal winds, while in the west, the city is influenced by the desert biome^52,53^. Sydney is also the most populated city in the country, currently hosting 5.3 million people^54^, and this figure is projected to increase to 8 million by 2053^47^. The city is adjacent to national parks in the north and south, therefore, urban expansion is largely occurring in the west. The greater Western Sydney region is projected to accommodate more than 50% of the greater Sydney region’s population by 2036^55^.

### Meteorological stations and data processing

In several studies, the association between UO and synoptic-scale weather conditions was investigated by using one urban and rural stations for ΔT calculation^17,26,27^. One meteorological station may not represent the whole geographical extent of the city. In the present study, Sydney was stratified into three zones: Western Sydney, inner Sydney, and eastern Sydney based on the distance from the coast. The Observatory Hill (OBS Hill) in eastern Sydney was considered the reference station as proposed in several other studies^13,53^. OBS Hill is in the proximity of the coast and the Sydney central business district (CBD). Inner Sydney comprises of Olympic Park and Canterbury, whereas Western Sydney contains the Penrith Lakes, Campbelltown, and Liverpool (Figure S17, and Table S1).

The tree canopy cover was estimated to be higher for the Western Sydney sites (25–35%)^56^, compared to inner Sydney and the Sydney CBD where it was around 15–17%. The potentially plantable surfaces were also higher for the Western Sydney sites (40–50%), compared with inner Sydney (22–32%), and Sydney CBD (13%)^56^. The population density^54^ for the Western Sydney sites varied between 500 and 650 pop/km^2^, for inner Sydney between 2500 and 4000 pop/km^2^, and for Sydney CBD it was over 6000 pop/km^2^. The distance from the nearest coast is increasing from eastern to Western Sydney, and in Western Sydney, Penrith Lakes is 50 km away from the nearest coast, whereas other Western Sydney sites are at around 25 km distance from the nearest coast. The inner Sydney sites are 8–12 km away from the nearest coast, and the Sydney CBD is located near the coast (Figure S17).

The half-hourly temperature data at different Sydney sites were mainly obtained from the Australian Bureau of Meteorology (BOM) ^57^. Previously, the comparison between the local and global-scales phenomena was also made for a shorter period (1–2 years)^29,31^, which may provide inconsistent results. In the present study, ΔT is compared with synoptic-scale weather conditions from 1999 to 2017. For Campbelltown, the data was available from December 2006 onward, while for Liverpool, the data was obtained from the NSW government^58^, which was available from 2013 onward. Validation procedures, including range test, step test, persistent test, and relational test were applied to eliminate the null values and the outliers from the data^59^. The gaps were infilled by applying the linear interpolation and triangulation techniques (data from three nearest station). The hourly temperature averages were calculated from the semi-hourly data, and then ΔT was computed on an hourly basis.

## Methods

Previously, various methods of ΔT calculation were applied to find the association between UO and synoptic-scale circulations. For instance, daily synoptic-scale weather conditions were compared either with daily mean UO^17,25,32^, daily maximum UO^31^, or daily minimum UO^28,30,38^. In the present study, both daily max ΔT (UO), and daily minimum ΔT (UC) are compared with the daily synoptic-scale weather conditions to find both extremes instead of averages as performed in several other studies^26,27^. Previously, ΔT was also computed with both ambient temperature^17,31,32^and the surface temperatures^26^. However, thermal comfort at 2 m height is more critical, thus, ΔT at 2 m height is computed. The circulation-to-environment^26,27,32^, and environment-to-circulation^18^, both approaches have been utilized to find an association between local and global-scales phenomena. In the present study, circulation-to-environment was the primary method of application as ΔT was organized according to daily synoptic-scale conditions. However, for UO, and UC comparison, the environment to circulation approach is also employed. ΔT might be computed either as an urban–rural temperature difference (UHI) or as an inner-urban temperature contrast^60^. Since, daytime temperatures are high in the surrounding suburbs, compared to Sydney CBD, therefore ΔT is calculated as (ΔT = T_suburb_ − T_urb_).

Initially, overall and seasonal synoptic-scale WTs frequency from 1999 to 2017 was investigated to understand which synoptic-scale conditions were occurring with the higher frequency. ΔT frequency at daytime and nighttime was examined to understand when UO and UC were occurring with higher frequency. Variations in ΔT magnitude over the years were also examined to understand the change in UO magnitude with respect to time. The higher 5% of daily maximum ΔT (UO), and lower 5% of daily minimum ΔT (UC) were compared with daily synoptic-scale conditions to comprehend, which conditions were primarily responsible for UO, and UC. The diurnal ΔT variations (daily daytime maximum ΔT, and daily nighttime minimum ΔT) were also associated with daily synoptic-scale weather conditions to validate the UO and UC results. The daytime and nighttime durations were defined according to sunrise and sunset. The seasonal variations in daily maximum ΔT were also linked to the daily synoptic-scale weather conditions. The association between daily synoptic-scale WTs and UO was also examined during the heatwave and non-heatwave episodes to understand the response of local phenomena during extreme heat events.

Note: The term UO is used instead of urban heat island (UHI) as instead of urban–rural temperature contrast, the thermal gradient is computed between various parts of the city. Further, the ΔT is computed as the temperature difference between suburban and urban sites (T_suburb_ – T _urb_) as the suburbs (inland sites) are thermally more affected and are becoming uninhabitable (particularly Western Sydney), compared to eastern suburbs/ Sydney CBD (coastal site) as reported in many other studies ^61^_′_^62^.

### Synoptic-scale classifications

The circulation-pattern-based classifications (CPC), and multivariate weather typing classification (WTC) both have been used to investigate the relationship between synoptic-scale circulations and different surface phenomena^33,63,64^. The WTC^65^ have proven to be more helpful, particularly in the studies related to the bioclimatology^66^, and the urban-climatology^36^. In the multivariate WTC, the spatial synoptic classification (SSC) has been widely used in finding the association between the synoptic-scale circulations, and the UO^27,28,32^. However, due to the human’s adaptability to the local climatic conditions, geographical and seasonal relative measures of the meteorological conditions are important features to be considered in the daily weather types. The SSC might not incorporate the seasonal and geographical relativity thoroughly^67^. However, such spatial–temporal relative conditions are considered in Gridded weather typing classification (GWTC)^68^. In the GWTC, the automated deseasonalized z-score initial typing procedure makes the character of airmass to occur at different locations in all seasons. Thus, all WTs may occur in all seasons/months, which minimize the seasonal frequency variability. Additionally, the character of the same airmass may also vary according to season and geographical location. In the present research, it is the first time that GWTC is being utilized to find an association between ΔT and daily synoptic-scale weather conditions. The GWTC data for Sydney was obtained from (https://www.personal.kent.edu/~cclee/gwtc2global.html).

### Gridded weather type classification (GWTC)

SSC was classified into seven weather types (WT): dry polar (DP), dry moderate (DM), dry tropical (DT), moist polar (MP), moist moderate (MM), moist tropical (MT), and transitional (TR)^42^. In the GWTC, days are classified into 11 WTs (nine core WTs, and two transitional WTs) by using the six near-surface weather variables (temperature, dew point temperature, wind speed, wind direction, mean sea level pressure, and cloudiness)^68^. The humid cold (HC), humid warm (HW), dry cool (DC), and dry warm (DW) are the corner/extreme WTs, more intense/ weak in terms of humidity and temperatures for a specific location, and time of the year. The humid (H), dry (D), cool (C), and warm (W) are moderate WTs, either in terms of temperature or the humidity for a specific location and time of the year. The seasonal (S) WT are moderate in terms of both temperature, and humidity for a specific location and time of the year. The cold frontal passage (CFP), and warm frontal passage (WFP) are the transitional WTs. Further details about the GWTC-WTs are provided in Table 2. The degree of the partitioning of the two variables (temperature, and humidity) among the WTs varies by region and season.

**Table 2:** Definition and characteristics of the WTs in GWTC.

Sr No.	Category	Type	Abbreviation	Description
1	Corner WTs	Humid cool	HC	Cooler and more humid than normal for the location and time of year
2		Humid warm	HW	Warmer and more humid than normal for the location and time of year
3		Dry cool	DC	Cooler and drier than normal for the location and time of year
4		Dry warm	DW	Warmer and drier than normal for the location and time of year
5	WTs moderate in terms of temperature	Humid	H	More humid than normal for the location and time of year
6		Dry	D	Drier than normal for the location and time of year
7	WTs moderate in terms of humidity	Cool	C	Cooler than normal for the location and time of year
8		Warm	W	Warmer than normal for the location and time of year
9	WT moderate in terms of both temperature and humidity	Seasonal	S	Near-normal conditions for the location and time of year
10	Transitional WTs	Cold front passage	CFP	Transitional weather day often with a drop in temperatures and dew points and rising sea-level pressure
11		Warm front passage	WFP	Transitional weather day often with increasing temperatures and dew points and lowering sea-level pressure

### Heatwave definition and selected heatwave periods

The UO response to daily synoptic-scale WTs was also investigated during heatwaves and non-heatwaves. Heatwave definition was applied as provided in research^14^, where the 95th percentile of daily maximum temperature was taken as the threshold temperature, occurring for three or more consecutive days. The spatial extension was also taken into account by considering only common heatwave periods at all sites in three zones of Sydney. The equal number of non-heatwave days were considered either before or after the heatwave episode to define the non-heatwave period. In total, ten heatwave and non-heatwave episodes were considered as shown in Table 3.

**Table 3:** Selected HW and NHW periods from 1999 to 2017 at all studied sites.

#	Year	Selected HW periods	Selected NHW periods
1	2000	24–26 Dec	28–30 Dec
2	2001	23–26 Jan	28–31 Jan
3	2004	12–14 Oct	16–18 Oct
4	2005	30 Dec–01 Jan	4–6 Jan 2006
5	2009	20–22 Nov	23–25 Nov
6	2011	01–05 Feb	7–10 Feb
7	2015	18–20 Nov	15–17 Nov
8	2016	19–21 Jan	22–24 Jan
9	2017	4–6 Feb	1–3 Feb
10	2017	9–11 Feb	13–15 Feb
Total days		33	32

## Supplementary Information

Supplementary Information.
