# Supplementary material for: Spatiotemporal variation in urban overheating magnitude and its association with synoptic air-masses in a coastal city
Source: Sci Rep. 2021 Mar 24;11:6762. doi: 10.1038/s41598-021-86089-2 (PMC7991413; doi:10.1038/s41598-021-86089-2)
Supplement: Supplementary file 1 — Supplementary Information. [file 41598_2021_86089_MOESM1_ESM.docx]

**Supplementary Information**

**Spatiotemporal variation in urban overheating magnitude and its association with synoptic air- masses in a coastal city.**

Hassan Saeed Khan^1, 3,^ *, Mat Santamouris^1^, Pavlos Kassomenos^2^, Riccardo Paolini^1^, Peter Caccetta^3^, Ilias Petrou^2^

^1^ School of Built Environment, University of New South Wales (UNSW), SYDNEY NSW 2052, Australia.

^2^ Department of Physics, University of Ioannina, GR-45110, Ioannina, Greece.

^3^ Data-61, [The Commonwealth Scientific and Industrial Research Organization](https://www.researchgate.net/institution/The_Commonwealth_Scientific_and_Industrial_Research_Organisation) (CSIRO), Dick Perry Ave, Kensington, Perth, WA 6151, Australia

*****Correspondence: [hassan.khan@unsw.edu.au](mailto:hassan.khan@unsw.edu.au)


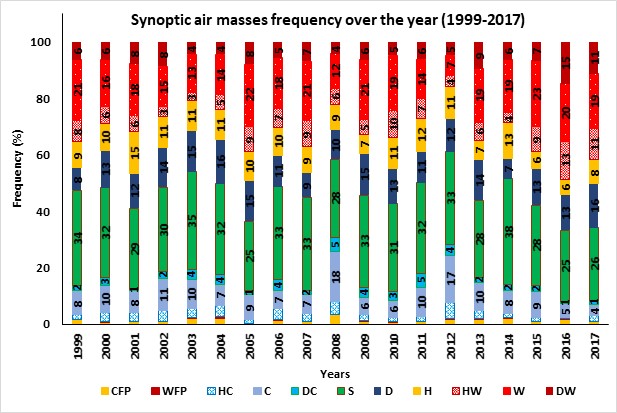


**Figure S1:** Synoptic air-masses frequencies over the year (1999-2017)

**
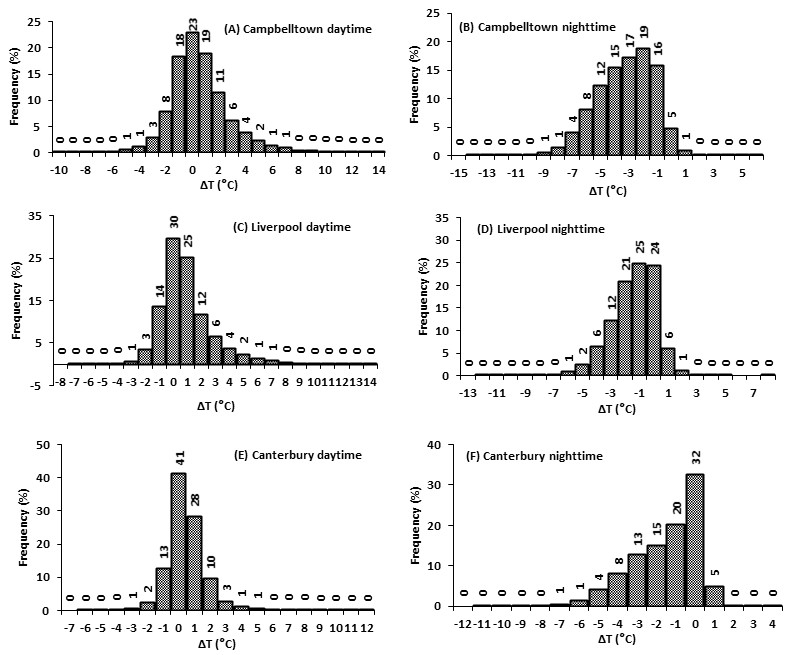
**

**Figure S2:** ΔT frequency distribution at different sites in Sydney at daytime and nighttime **A)** Campbelltown at **daytime**, **B)** Campbelltown at **nighttime**, **C)** Liverpool at **daytime**, **D)** Liverpool at **nighttime**. **C)** Canterbury at **daytime**, **D)** Canterbury at **nighttime**.


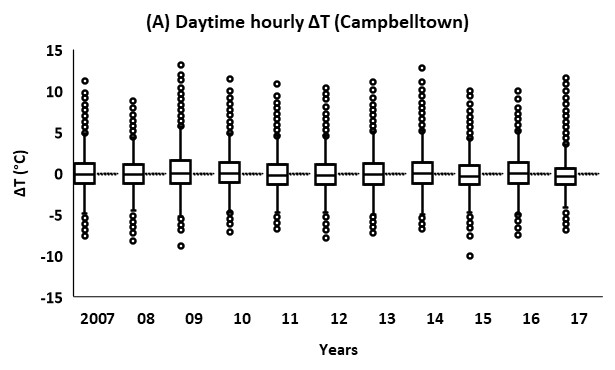

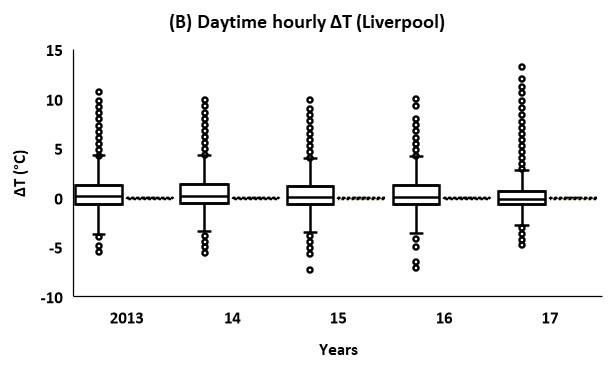

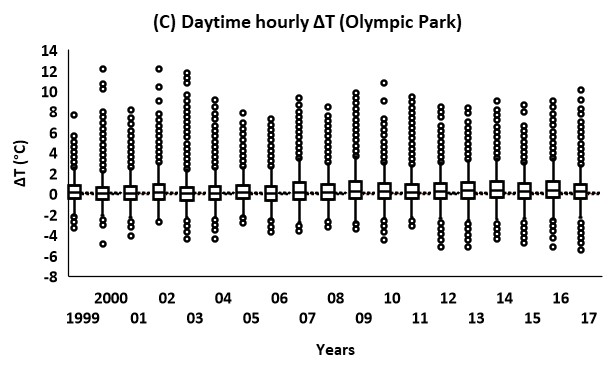

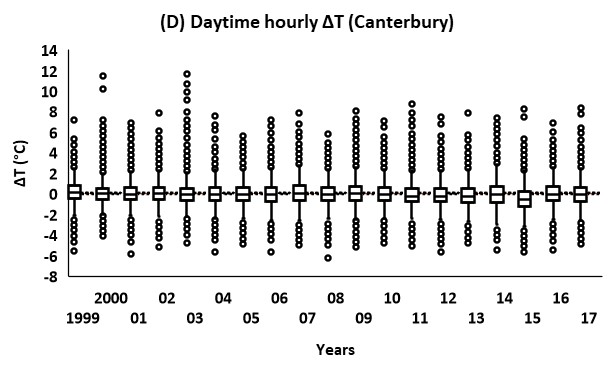


**Figure S3:** Variations in **daytime hourly ΔT** over time (1999-2017) at various Sydney sites, **A)** Campbelltown, **B)** Liverpool, **C)** Olympic Park, **D)** Canterbury. **ΔT=0** is illustrated by a dotted line. The boxplots are plotted according to the general convention **(**upper and lower extremes: **Q1-1.5*IQR and Q3+1.5*IQR).**


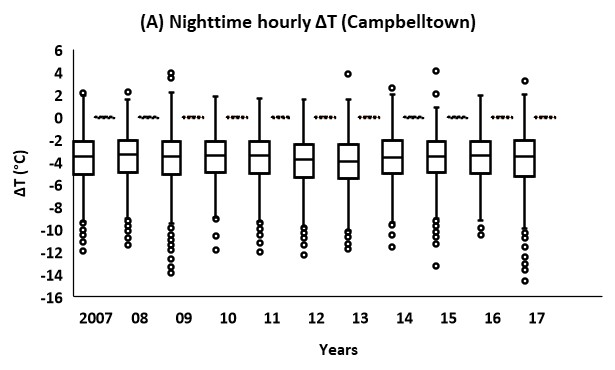

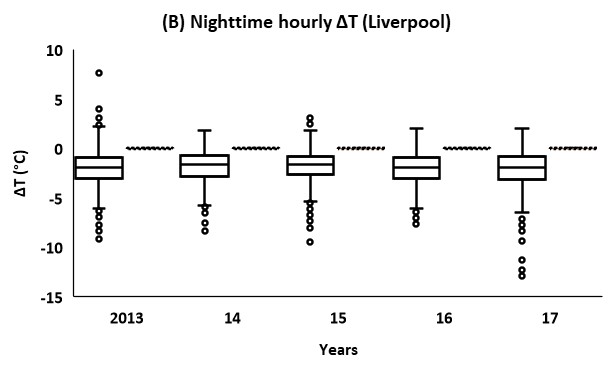

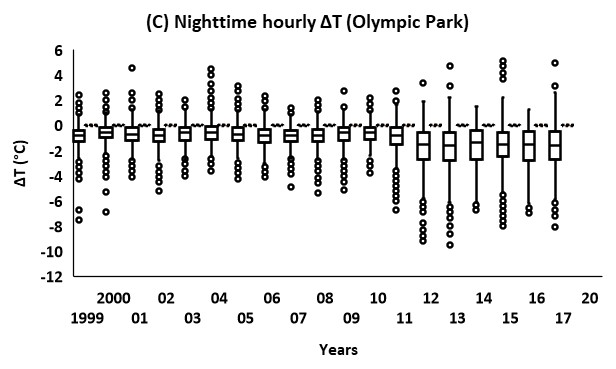

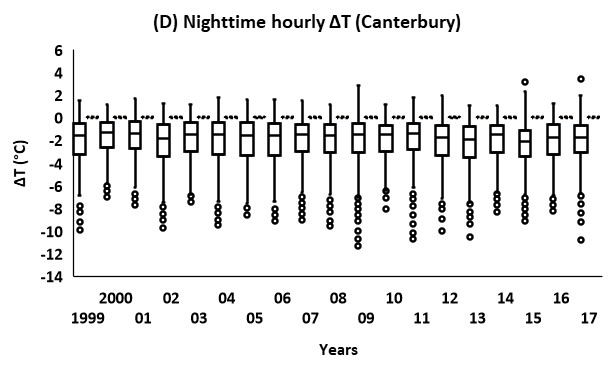


**Figure S4:** Variations in **nighttime hourly ΔT** over time (1999-2017) at various Sydney sites, **A)** Campbelltown, **B)** Liverpool, **C)** Olympic Park, **D)** Canterbury. **ΔT=0** is illustrated by a dotted line. The boxplots are plotted according to the general convention **(**upper and lower extremes: **Q1-1.5*IQR and Q3+1.5*IQR).**


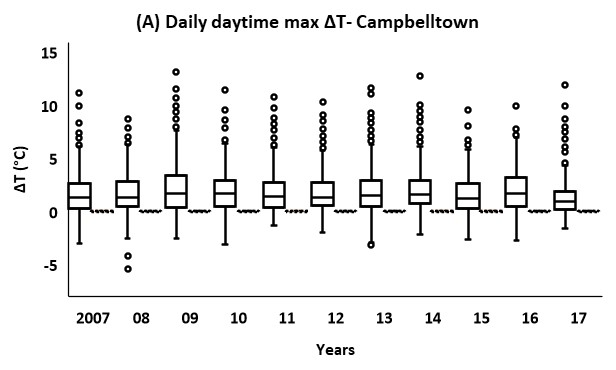

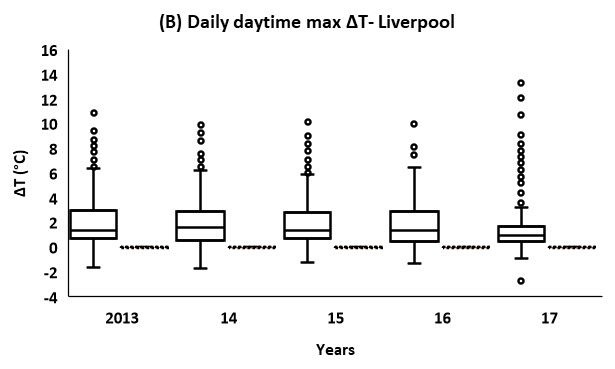

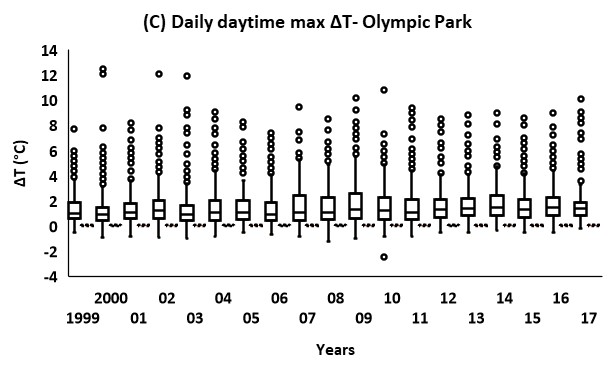

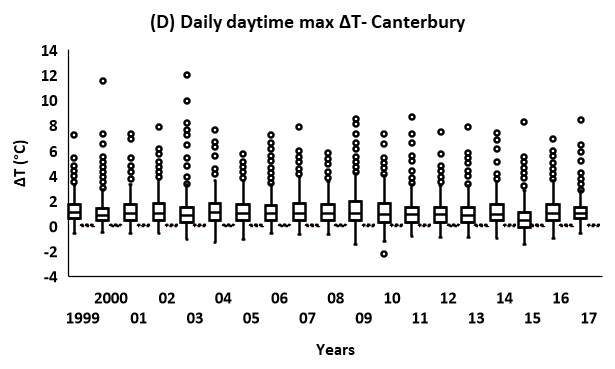


**Figure S5:** Variations in **daily max ΔT** over time (1999-2017) at various Sydney sites, **A)** Campbelltown, **B)** Liverpool, **C)** Olympic Park, **D)** Canterbury. **ΔT=0** is illustrated by a dotted line. The boxplots are plotted according to the general convention **(**upper and lower extremes: **Q1-1.5*IQR and Q3+1.5*IQR).**


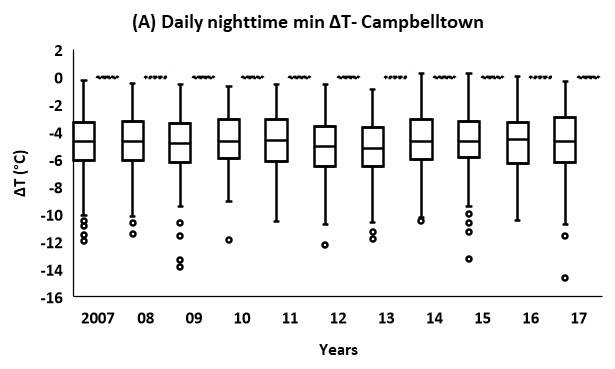

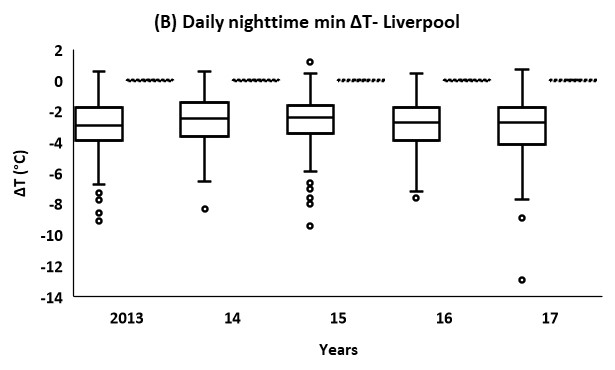

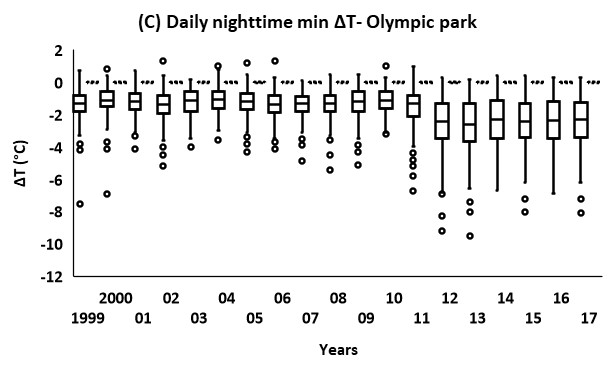

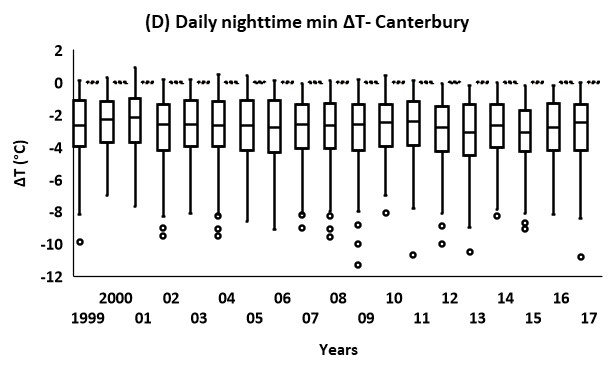


**Figure S6:** Variations in **daily min ΔT** over time (1999-2017) at various Sydney sites, **A)** Campbelltown, **B)** Liverpool, **C)** Olympic Park, **D)** Canterbury. **ΔT=0** is illustrated by a dotted line. The boxplots are plotted according to the general convention **(**upper and lower extremes: **Q1-1.5*IQR and Q3+1.5*IQR).**


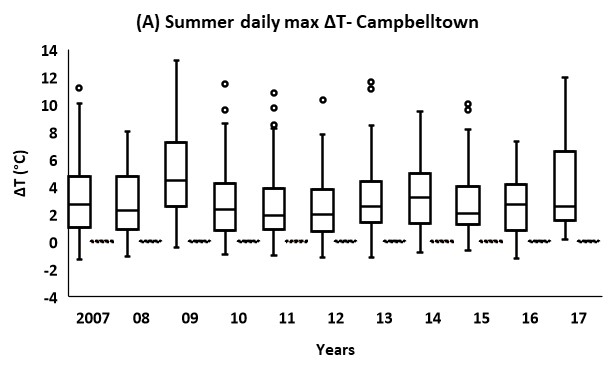

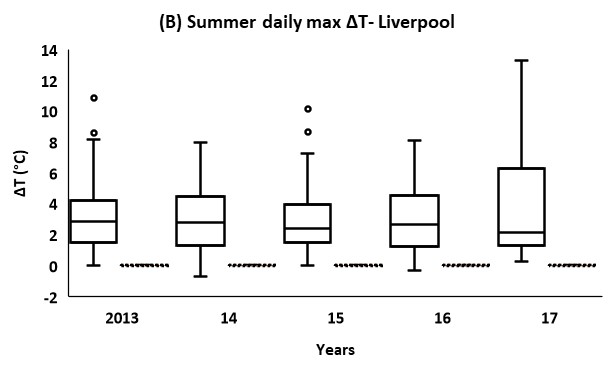

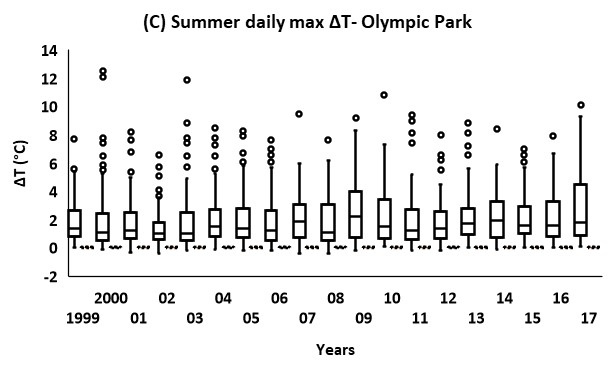

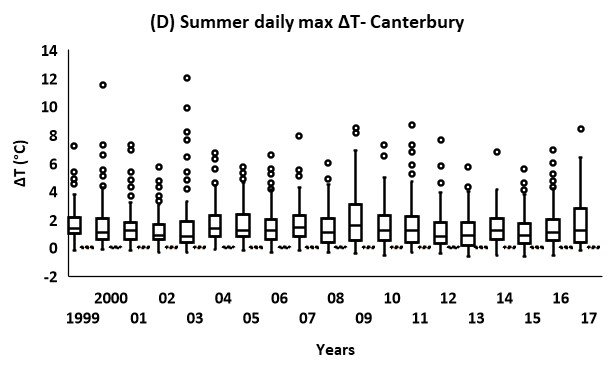


**Figure S7:** Variations in **Summer daily max ΔT** over time (1999-2017) at various Sydney sites, **A)** Campbelltown, **B)** Liverpool, **C)** Olympic Park, **D)** Canterbury. **ΔT=0** is illustrated by a dotted line. The boxplots are plotted according to the general convention **(**upper and lower extremes: **Q1-1.5*IQR and Q3+1.5*IQR).**


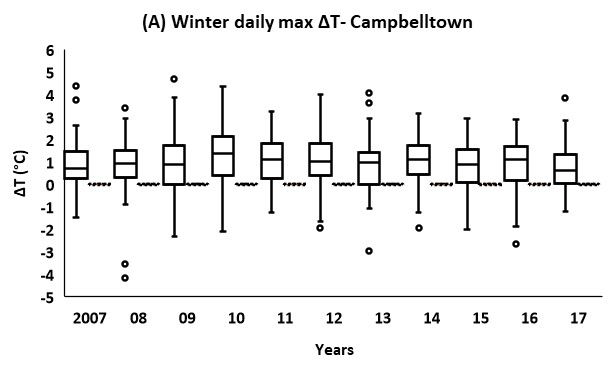

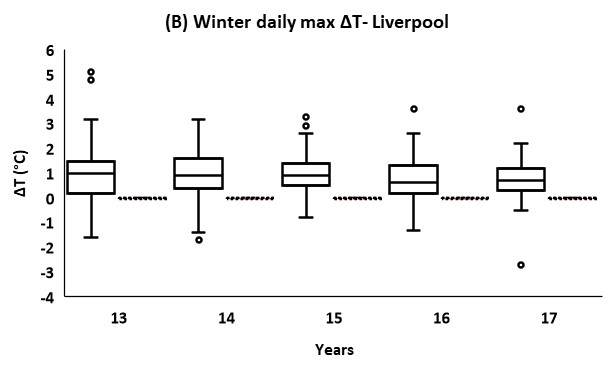

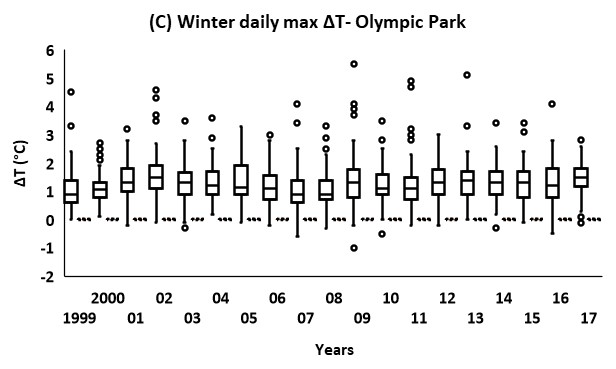

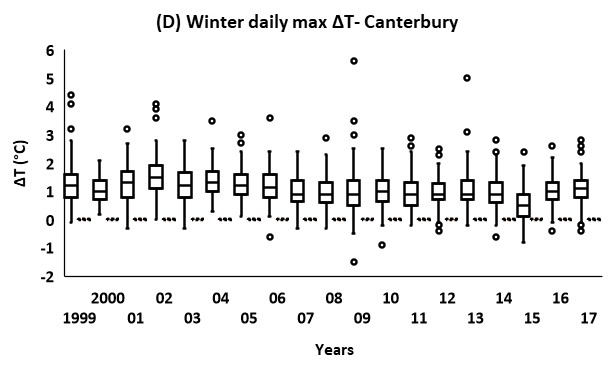


**Figure S8:** Variations in **Winter daily max ΔT** over the time (1999-2017) at various Sydney sites, **A)** Campbelltown, **B)** Liverpool, **C)** Olympic Park, **D)** Canterbury. **ΔT=0** is illustrated by a dotted line. The boxplots are plotted according to the general convention **(**upper and lower extremes: **Q1-1.5*IQR and Q3+1.5*IQR).**


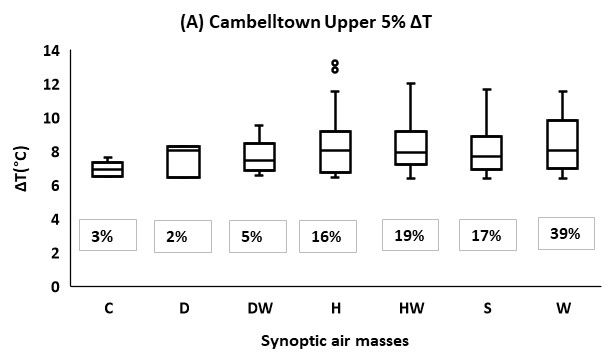

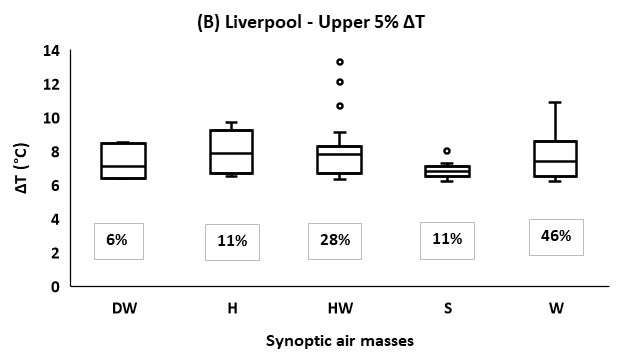

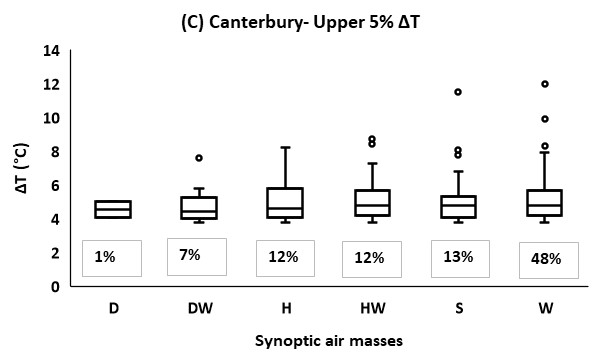

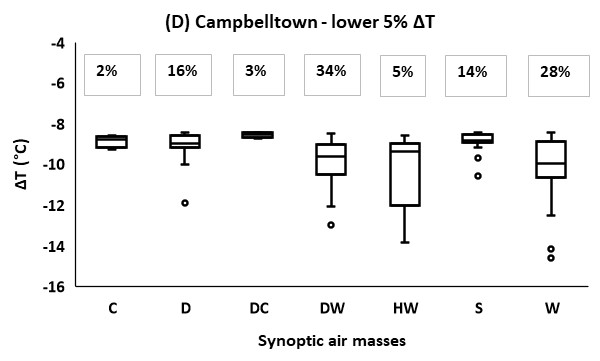

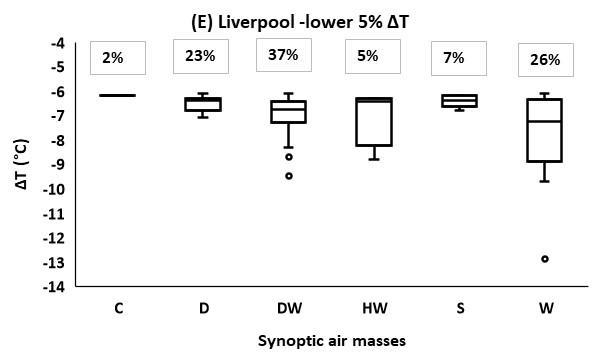

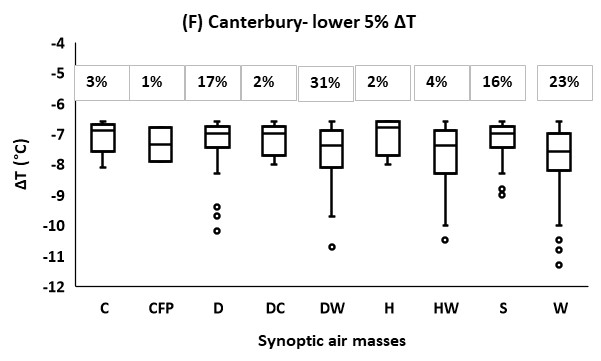


**Figure S9:** Higher 5% (UO) and lower 5% (UC) ΔT at different Sydney sites under different synoptic air-masses. **A)** **UO** at Campbelltown, **B)** **UO** at Liverpool, **C)** **UO** at Canterbury, **D)** **UC** at Campbelltown, **E)** **UC** at Liverpool, **F)** **UC** at Canterbury. The boxplots are plotted according to the general convention **(**upper and lower extremes: **Q1-1.5*IQR and Q3+1.5*IQR).**


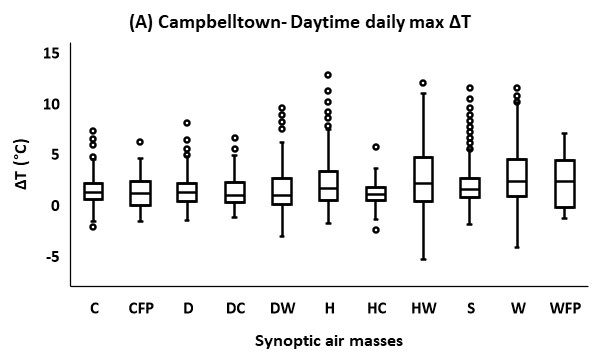

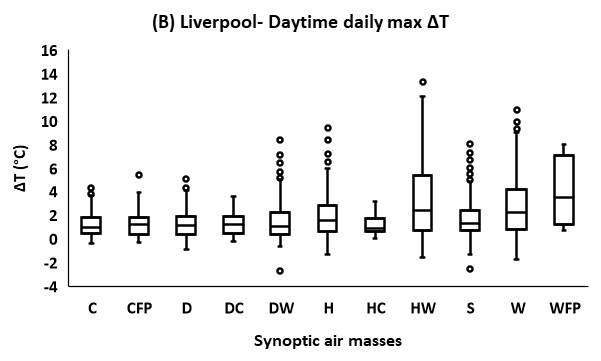

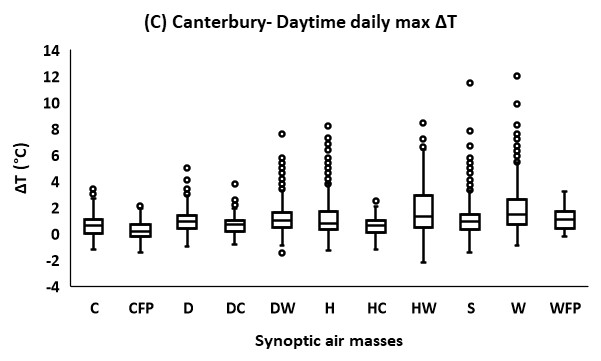

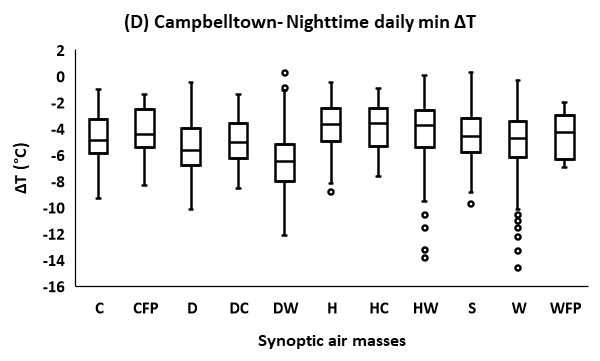

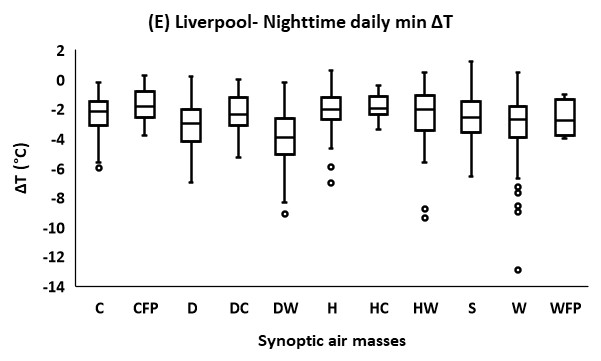

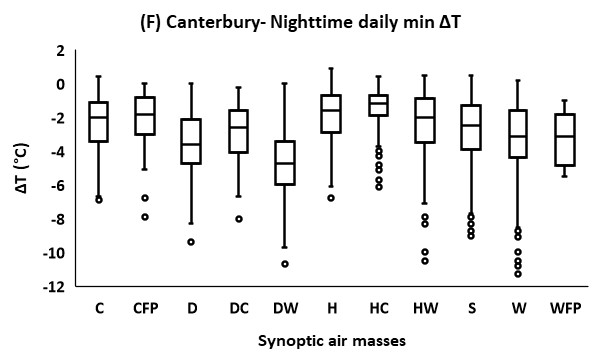


**Figure S10:** Daytime daily maximum ΔT and nighttime daily minimum ΔT at different Sydney sites under different synoptic air-masses. **A)** **Daytime** daily max ΔT at Campbelltown, **B)** **Daytime** daily max ΔT at Liverpool, **C)** **Daytime** daily max ΔT at Canterbury, **D)** **Nighttime** daily minimum ΔT at Campbelltown, **E)** **Nighttime** daily minimum ΔT at Liverpool, **F)** **Nighttime** daily minimum ΔT at Canterbury. The boxplots are plotted according to the general convention **(**upper and lower extremes: **Q1-1.5*IQR and Q3+1.5*IQR).**


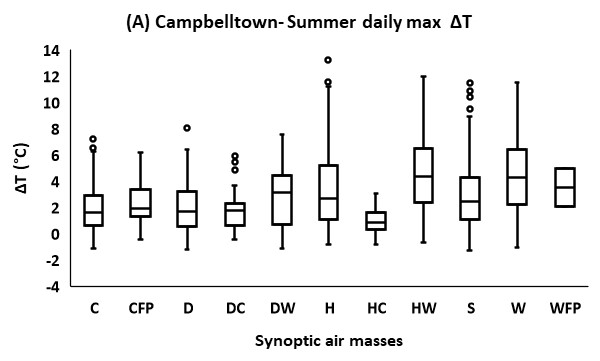

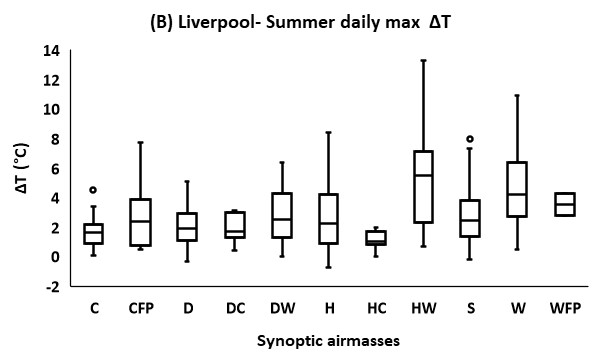

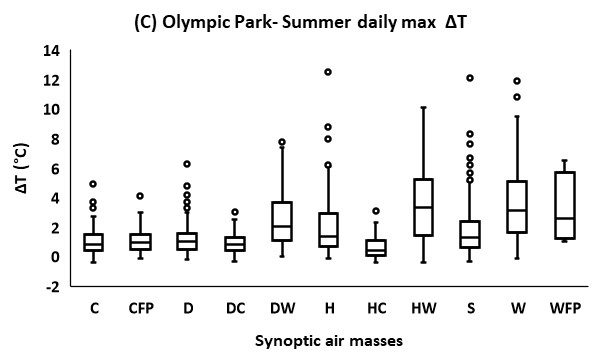

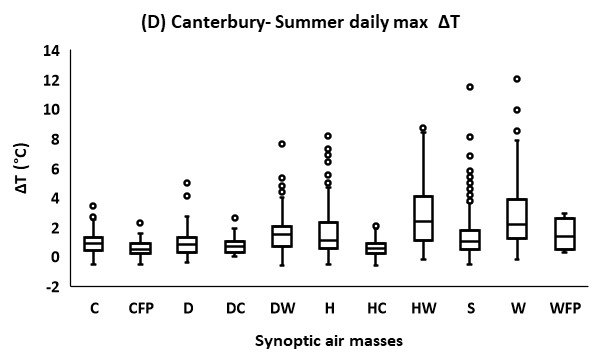


**Figure S11:** Synoptic air-masses and daily max ΔT comparison during **summer** (1999-2017), **A)** Campbelltown, **B)** Liverpool, **C)** Olympic Park, **D)** Canterbury. The boxplots are plotted according to the general convention **(**upper and lower extremes: **Q1-1.5*IQR and Q3+1.5*IQR).**


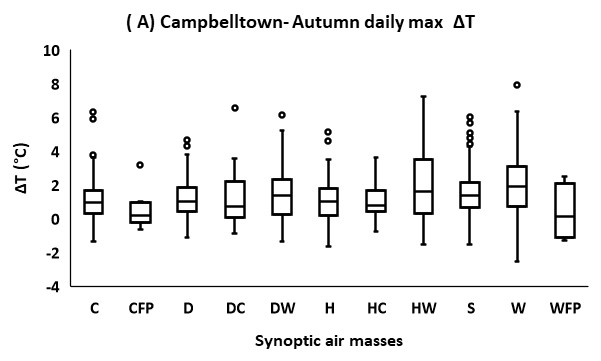

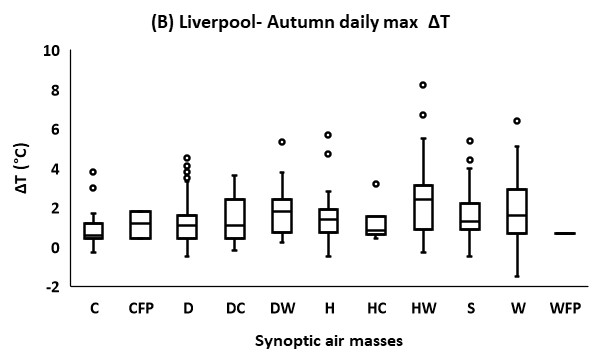

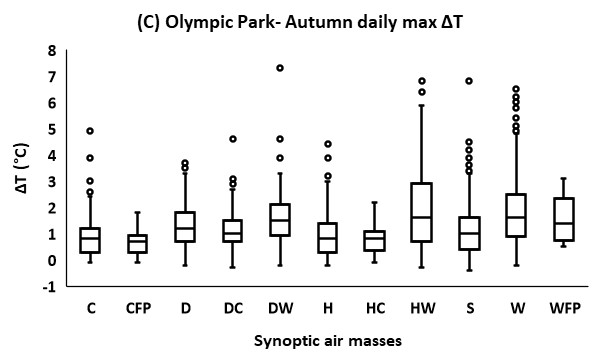

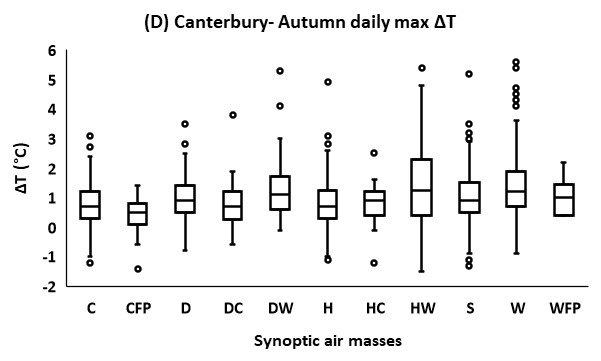


**Figure S12:** Synoptic air-masses and daily max ΔT comparison during **Autumn** (1999-2017), **A)** Campbelltown, **B)** Liverpool, **C)** Olympic Park, **D)** Canterbury. The boxplots are plotted according to the general convention **(**upper and lower extremes: **Q1-1.5*IQR and Q3+1.5*IQR).**


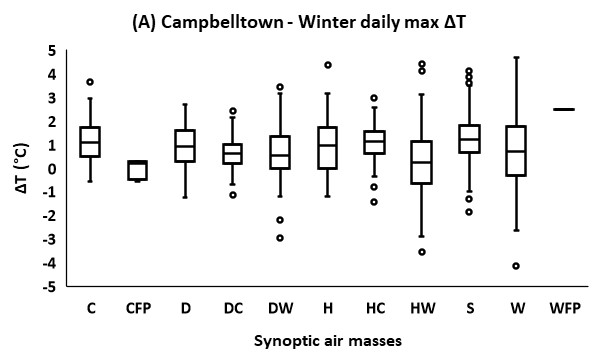

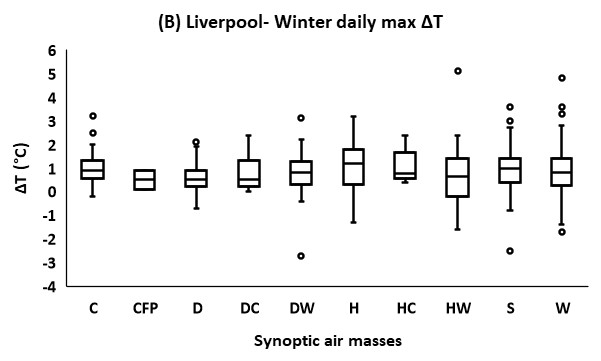

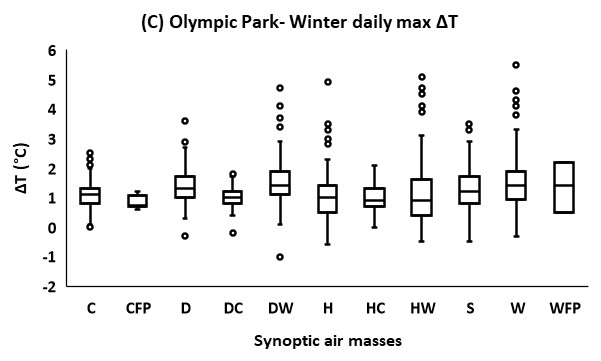

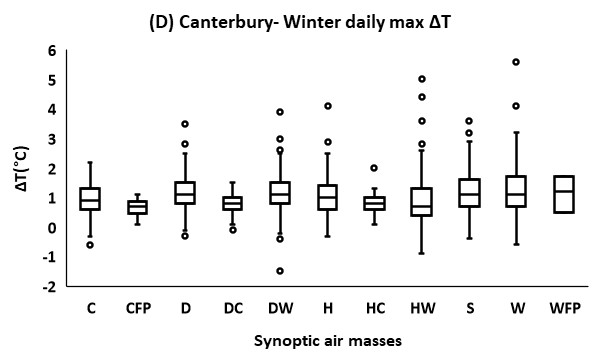


**Figure S13:** Synoptic air-masses and daily max ΔT comparison during **Winter** (1999-2017), **A)** Campbelltown, **B)** Liverpool, **C)** Olympic Park, **D)** Canterbury. The boxplots are plotted according to the general convention **(**upper and lower extremes: **Q1-1.5*IQR and Q3+1.5*IQR).**


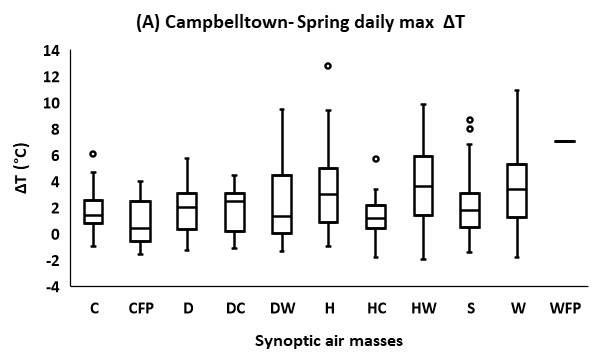

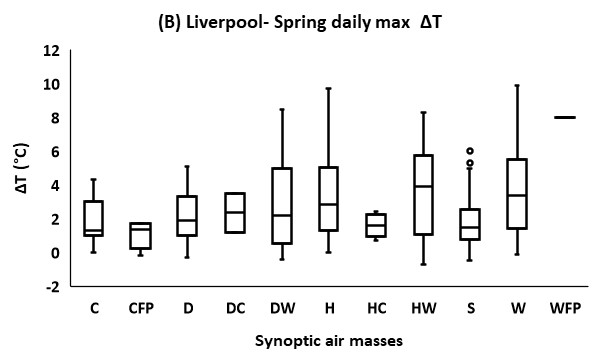

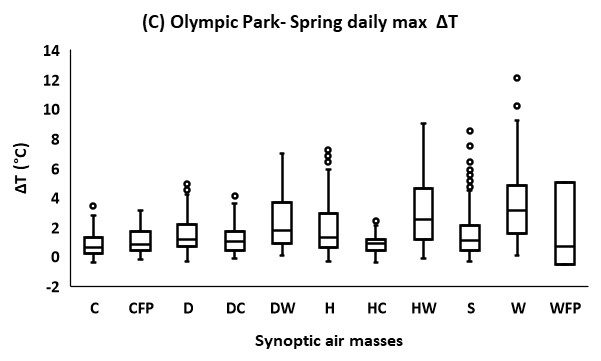

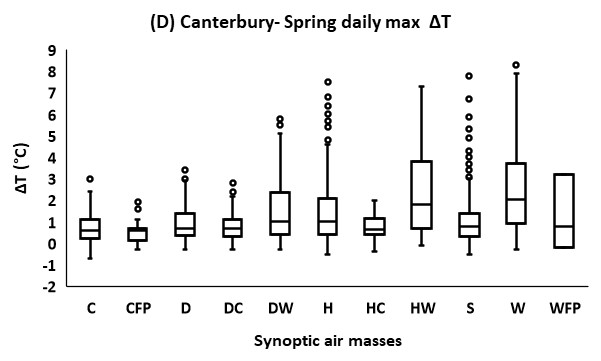


**Figure S14:** Synoptic air-masses and daily max ΔT comparison during **Spring** (1999-2017), **A)** Campbelltown, **B)** Liverpool, **C)** Olympic Park, **D)** Canterbury. The boxplots are plotted according to the general convention **(**upper and lower extremes: **Q1-1.5*IQR and Q3+1.5*IQR).**


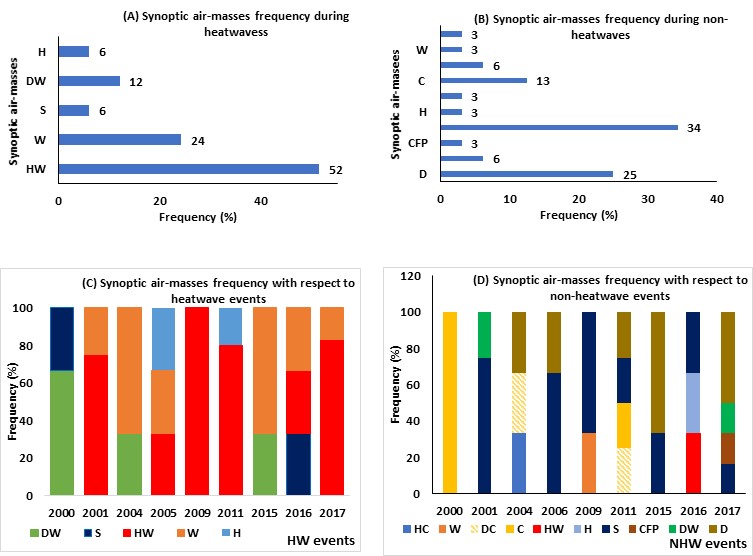


**Figure S15:** Synoptic air-masses frequency during heatwaves and non-heatwaves. **A)** overall during heatwaves, **B)** Overall during non-heatwaves, **C)** Event-wise during heatwaves, **D)** Event-wise during non-heatwaves.


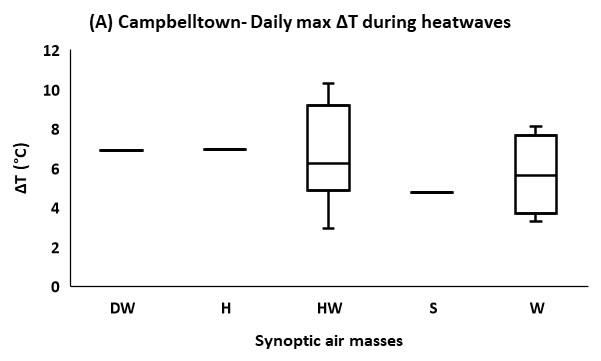

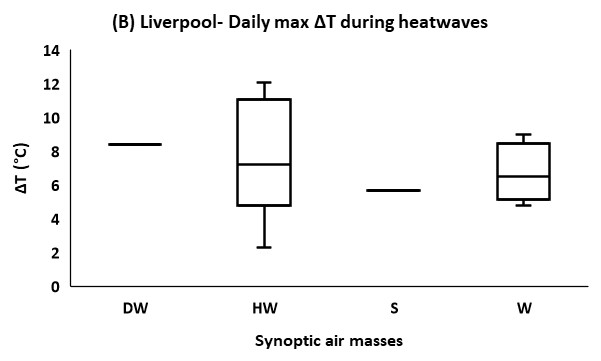

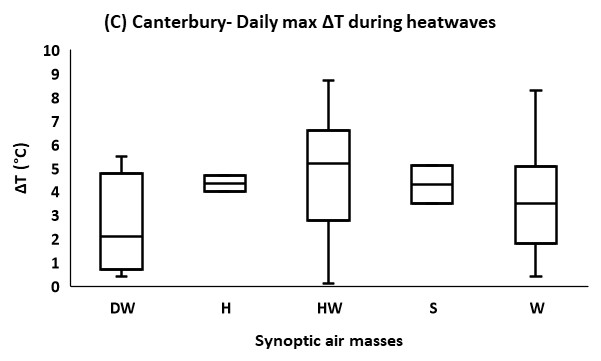

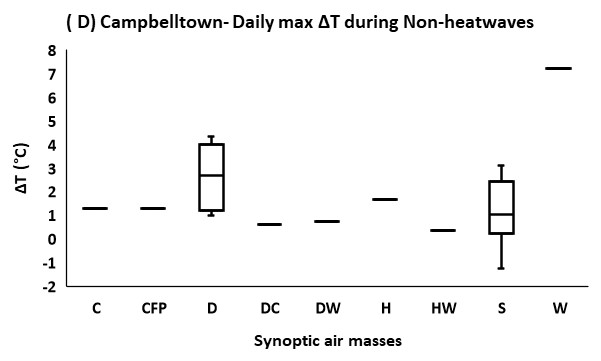

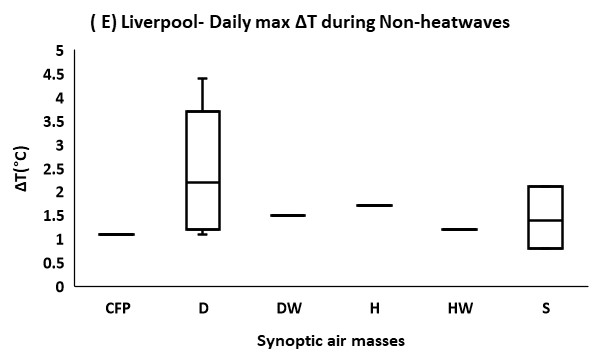

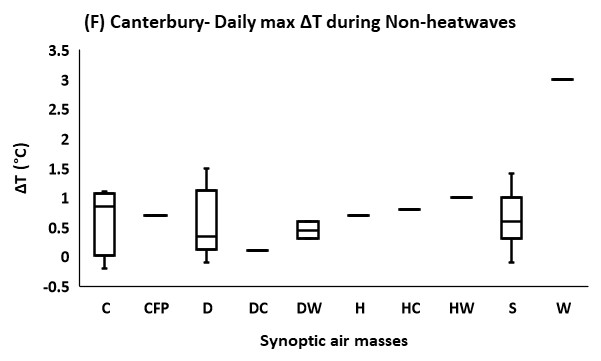


**Figure S16:** Synoptic air-masses and *daily max* ΔT during heatwaves and Non-heatwaves). **A)** Campbelltown during **heatwaves**, **B)** Liverpool during **heatwaves**, **C)** Canterbury during **heatwaves D)** Campbelltown during **Non-heatwaves**, **E)** Liverpool during **Non-heatwaves**, **F)** Canterbury during **Non-heatwaves.** The boxplots are plotted according to the general convention **(**upper and lower extremes: **Q1-1.5*IQR and Q3+1.5*IQR).**


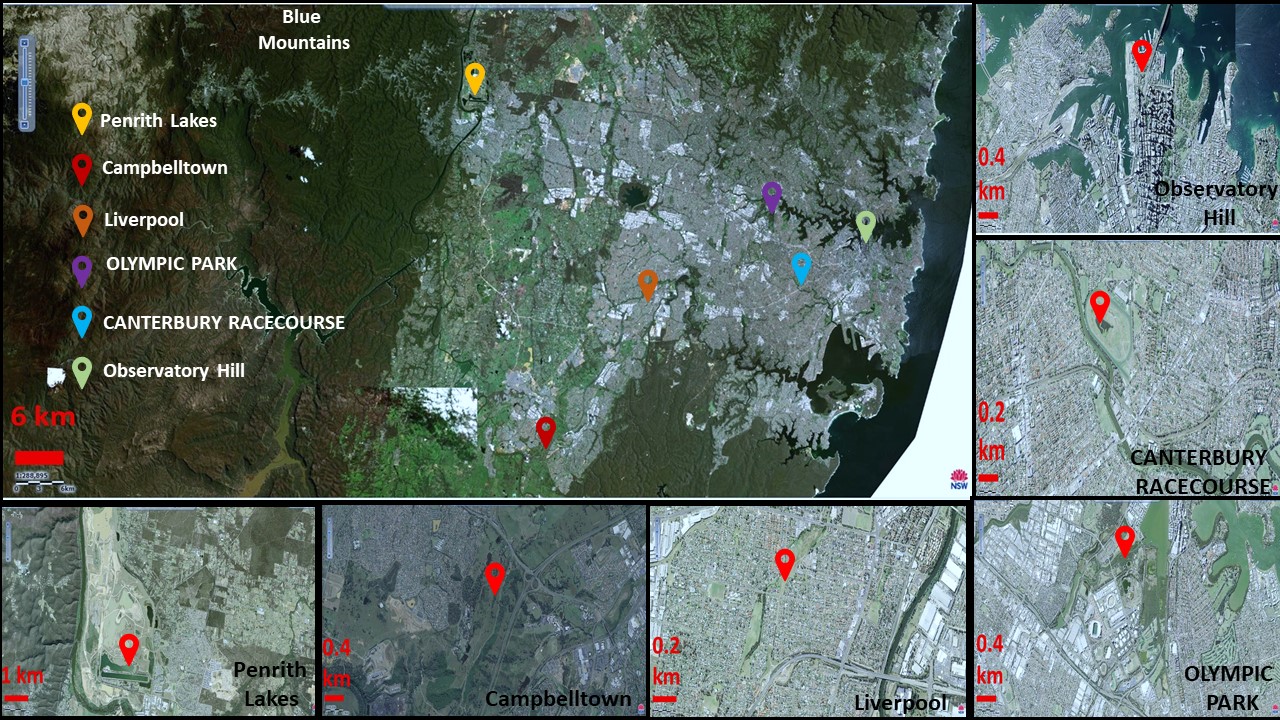


**Figure S17:** Location of weather stations, and site characteristics. **Image source:** NSW government (<https://maps.six.nsw.gov.au/>) under copyright license (<https://www.spatial.nsw.gov.au/copyright>) Creative Commons Australia Attribution 4.0 license (<http://creativecommons.org/licenses/by-nd/4.0>). Note: Location markers, site-scale, and the legend have been added by utilizing the MS PowerPoint to make the information legible.

**Table S1:** Geographical information and description of weather stations.

| **Sr No.** | **Station** | **Zone** | **Latitude** | **Longitude** | **Station Height (m)** | **Land use** |
| --- | --- | --- | --- | --- | --- | --- |
| 1 | Observatory Hill (OBS Hill) | Eastern Sydney | 33°51'39"S | 151°12'18"E | 39 | Dense Commercial |
| 2 | Canterbury Racecourse | Inner Sydney | 33°54'21"S | 151°6'48"E | 3 | Residential/ industrial |
| 3 | Olympic Park |  | 33°50'2"S | 151°4'18"E | 4 | Mixed used |
| 4 | Liverpool | Western Sydney | 33° 55' 58"S | 150° 54' 21"E | 22 | Residential/ commercial |
| 5 | Campbelltown |  | 34°3'41"S | 150°46'25"E | 112 | Residential/ semi-rural |
| 6 | Penrith Lakes |  | 33°43'10"S | 150°40'42"E | 24.7 | Residential/ commercial |
